# MKL1-actin pathway restricts chromatin accessibility and prevents mature pluripotency activation

**DOI:** 10.1038/s41467-019-09636-6

**Published:** 2019-04-12

**Authors:** Xiao Hu, Zongzhi Z. Liu, Xinyue Chen, Vincent P. Schulz, Abhishek Kumar, Amaleah A. Hartman, Jason Weinstein, Jessica F. Johnston, Elisa C. Rodriguez, Anna E. Eastman, Jijun Cheng, Liz Min, Mei Zhong, Christopher Carroll, Patrick G. Gallagher, Jun Lu, Martin Schwartz, Megan C. King, Diane S. Krause, Shangqin Guo

**Affiliations:** 1 Department of Cell Biology, Yale University, New Haven, CT 06520 USA; 2 Yale Stem Cell Center, Yale University, New Haven, CT 06520 USA; 3 Department of Pathology, Yale Cancer Center, Yale University, New Haven, CT 06520 USA; 4 Department of Pediatrics, Yale University, New Haven, CT 06520 USA; 5 Department of Medicine (Cardiology), Yale University, New Haven, CT 06520 USA; 6 Department of Genetics, Yale University, New Haven, CT 06520 USA; 7 Department of Laboratory Medicine, Yale University, New Haven, CT 06520 USA

## Abstract

Actin cytoskeleton is well-known for providing structural/mechanical support, but whether and how it regulates chromatin and cell fate reprogramming is far less clear. Here, we report that MKL1, the key transcriptional co-activator of many actin cytoskeletal genes, regulates genomic accessibility and cell fate reprogramming. The MKL1-actin pathway weakens during somatic cell reprogramming by pluripotency transcription factors. Cells that reprogram efficiently display low endogenous MKL1 and inhibition of actin polymerization promotes mature pluripotency activation. Sustained MKL1 expression at a level seen in typical fibroblasts yields excessive actin cytoskeleton, decreases nuclear volume and reduces global chromatin accessibility, stalling cells on their trajectory toward mature pluripotency. In addition, the MKL1-actin imposed block of pluripotency can be bypassed, at least partially, when the Sun2-containing linker of the nucleoskeleton and cytoskeleton (LINC) complex is inhibited. Thus, we unveil a previously unappreciated aspect of control on chromatin and cell fate reprogramming exerted by the MKL1-actin pathway.

## Abstract (web summary)

MKL1 is a key transcriptional co-activator of actin cytoskeleton genes. Here the authors show that MKL1 activation in somatic cells reduces chromatin accessibility and hinders full reprogramming to pluripotency. Reduction of MKL1, disruption of actin cytoskeleton and its links to the nucleus relieve this repression.

## Introduction

The nucleus orchestrates characteristic gene expression programs often by modulating chromatin accessibility, thereby determining cellular identity. Chromatin accessibility is best known to be catalyzed by biochemical activities from various nuclear-localized epigenetic remodeling enzymes^1,2^. Whether the nucleus and chromatin accessibility is controlled by elements external to the nucleus, such as those conducting the biomechanical cues, is largely unexplored.

The nucleus is physically connected with the cytoskeleton via the linker of the nucleoskeleton and cytoskeleton (LINC) complex, a highly conserved nuclear envelope bridge consisting of Sun proteins and Nesprins^3–5^. It is known that the cytoskeleton and the LINC system are responsible for physically positioning the nucleus inside the cell and for deforming it in response to mechanical signals^6–9^. Mechanical strains on the nucleus mediated by the actomyosin system could be severe enough to cause nuclear envelope herniation or rupture^7,10–12^. Strains from polymerized actins have also been reported to cause transcriptional repression^13^. These evidences suggest that in addition to regulating the physical state of the nucleus, the cytoskeleton might also be able to modify the nucleus’ biochemical state. However, the extent and nature of this modulation, as well as the underlying mechanism remain unclear.

We explored these questions using somatic cell reprogramming into pluripotency as a model system. Pluripotent stem cells display highly open/accessible chromatin^14,15^, which can be experimentally induced from somatic cells of much reduced genomic accessibility. During reprogramming, when the transcription factors Oct4/Sox2/Klf4 (OSK) are first expressed in fibroblasts, they fail to bind the authentic pluripotency sites even though they are considered to possess pioneer activity^16,17^. The promiscuous binding by these pioneer factors to the somatic genome suggests that chromatin accessibility might be initially constrained by mechanisms that are particularly active in somatic cells. Here, we report that the actin cytoskeleton, and the main transcription factor complex controlling its abundance, MKL1/SRF, limits cell fate reprogramming by regulating global chromatin accessibility. High MKL1 activity generates excessive actins, polymerization of which leads to a significantly reduced nuclear volume via a mechanism involving the LINC complex. Within the small nucleus, chromatin accessibility is impaired and endogenous pluripotency fails to establish. Overall, we propose that the actin cytoskeleton is capable of constraining global chromatin accessibility. The highly accessible pluripotent genome is accommodated by a weak actin cytoskeleton.

## Results

### Reprogramming is accompanied by reduced actin-MKL1 activity

Our previous work revealed that somatic cells with an ultrafast cell cycle are efficiently reprogrammed via ectopic expression of Oct4/Sox2/Klf4/Myc (OSKM), a property that allows for their prospective isolation^18^. The fast cycling cells were morphologically distinct as compared to their slower cycling counterparts (Supplementary Fig. 1a). While the slow cycling cells had a typical fibroblastic appearance, the fast cycling cells appeared light-reflective and minimally spread (Supplementary Fig. 1a). This morphological distinction suggests underlying differences in the level and/or conformation of their cytoskeletal components. Indeed, the fast cycling cells displayed reduced expression in many actin and related genes (Supplementary Fig. 1b), but not in tubulin genes (Supplementary Fig. 1c)^18^, revealing a specific correlation with the actin cytoskeletal system. Thus, we investigated the role of the actin-based cytoskeleton in reprogramming.

**Fig. 1:**
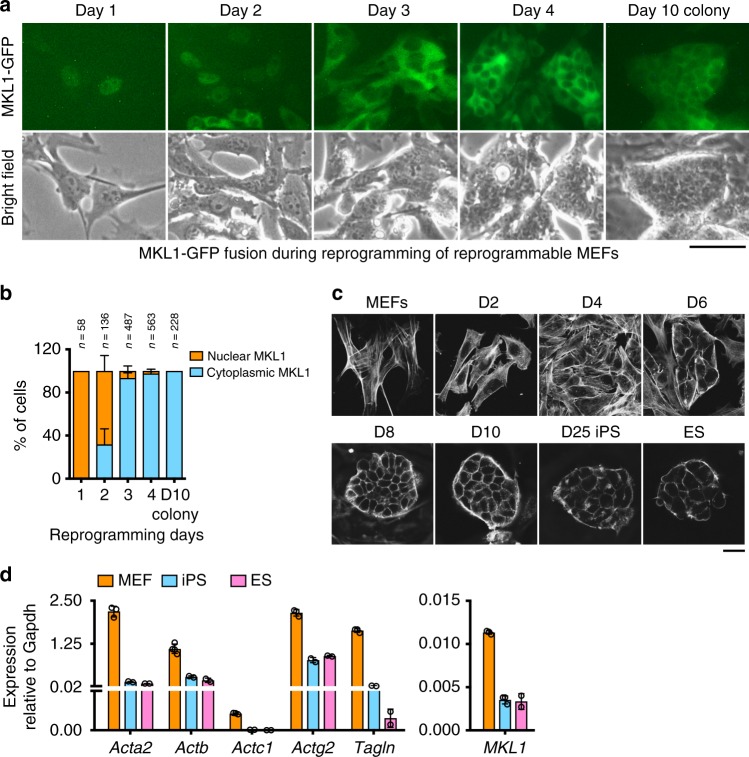
Somatic cell reprogramming to pluripotency is accompanied by reduced actin-MKL1 pathway activity. **a** Time-course imaging of MKL1-GFP localization in MEFs undergoing reprogramming. Representative images acquired daily (day 1–4) during early reprogramming and on day 10 for a colony with iPS-like morphology. Note MKL1-GFP gradually localizes to the cytoplasm as reprogramming progresses. Scale bar: 50 μm. **b** Quantification of the % of cells displaying nuclear- or cytoplasmic-localized MKL1-GFP in **a**. Images from three independent experiments were analyzed, and data were pooled from the indicated numbers of cells for each condition. **c** Representative confocal images of reprogramming cultures stained with phalloidin (F-actin) through the time course (MEF, day 2, day 4, day 6, day 8, day 10, and day 25). A typical ESC colony was stained as a control. Scale bar: 20 μm. **d** Realtime QPCR analyses of the mRNA levels of actin related genes, as well as MKL1 itself in MEFs, iPSCs and ESCs. Data are representative of three independent experiments. Error bars denote standard deviation of triplicate samples (*n* = 3) in each experiment

The expression of many actin cytoskeletal genes is controlled by the transcriptional co-activator, MKL1 (Megakaryoblastic Leukemia 1, MRTF-A), in complex with the Serum Response Factor (SRF) via binding to the CArG consensus sequence (Supplementary Fig. 1d)^19,20^. The transcriptional activity of MKL1 is primarily controlled by its cytoplasmic-nuclear shuttling via binding to monomeric actins^21^. To determine whether the subcellular localization of MKL1 changes during reprogramming, we tracked the localization of a virally expressed MKL1-GFP fusion protein^22^ in mouse embryonic fibroblasts (MEFs) undergoing reprogramming, during which all cells expressed OKSM when doxycycline (Dox) was added^23^. While many cells displayed nuclear MKL1-GFP at the onset of reprogramming, as expected for fibroblasts grown in the presence of serum^24^, its subcellular localization became predominantly cytoplasmic when reprogramming progressed (Fig. 1a, b), indicating reduced MKL1 activity. The altered MKL1 localization was paralleled by significant reduction in F-actins, as determined by phalloidin staining (Fig. 1c). At gene expression level, the resulting induced pluripotent stem cells (iPSCs) showed reduced expression of many actin cytoskeletal genes, as well as low levels of endogenous MKL1 as compared to MEFs (Fig. 1d). Of note, mouse embryonic stem cells (mESCs) displayed similarly low expression and polymerization of actins (Fig. 1c, d), suggesting that the MKL1-actin pathway activity is low in pluripotent cells. Overall, MKL1 activity and actin cytoskeletal genes are decreased during somatic cell reprogramming into pluripotency.

### MKL1 elevates cytoskeletal genes and blocks pluripotency

**Fig. 2:**
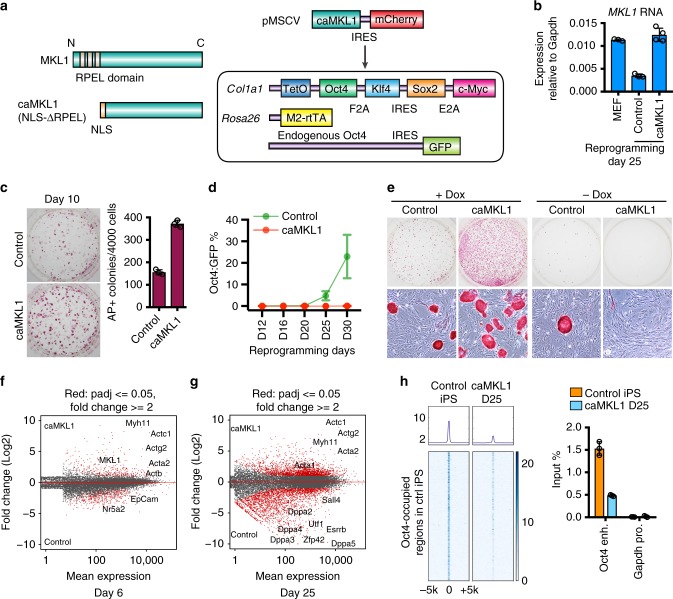
MKL1 drives actin cytoskeletal gene expression and blocks mature pluripotency. **a** Left: schematic diagram of constitutively active MKL1 (caMKL1), with the RPEL domain deleted and replaced with a nuclear localization signal (NLS). Right: experimental design illustrating a retroviral vector expressing caMKL1-IRES-mCherry to be transduced into reprogrammable MEFs, which express *Col1a:OKSM, Rosa26:M2rtTA* and *Oct4:GFP*. The co-expressed mCherry from the vector was used to isolate the transduced cells for all further experiments. **b** Realtime QPCR analysis of total MKL1 mRNA level in primary MEFs, as compared to the cells at reprogramming day 25 coexpressing either a control vector or caMKL1. *n* = 3 for MEF and control; *n* = 4 for caMKL1-overexpressing condition. Error bars denote standard deviation. **c** AP stained reprogramming cultures on day 10, coexpressing either control vector or caMKL1. The number of AP+ colonies derived from 4,000 reprogrammable MEFs are quantified. **d** Percentage of Oct4:GFP+ cells arising from reprogramming MEFs coexpressing control vector or caMKL1, as determined by FACS. Cells were trypsinized and analyzed at indicated time points starting from day 10. **e** AP stained reprogramming cultures from control- or caMKL1-expressing reprogramming MEFs on day25, when cells were passaged and replated into continued cultures with or without Dox. Bottom panel shows colonies at higher magnification. Note that no AP+ colonies could be detected in the caMKL1-expressing cultures without Dox. **f**, **g** MA plots of differentially expressed genes between control- and caMKL1-overexpressing cells at reprogramming day 6 (**f**) and day 25 (**g**), respectively. **h** Meta analysis of Oct4-bound regions in day 25 reprogramming cells expressing the control vector or caMKL1 (left). The Oct4-bound regions in control iPS cells show reduced Oct4 binding in caMKL1-expressing cells. Oct4 ChIP-qPCR confirmed reduced binding at the *Oct4* enhancer region in caMKL1-blocked cells (right). Error bars denote standard deviation of triplicate samples (*n* = 3) in each one of three independent experiments (**c**, **d**, **h**)

To test whether the reduction in actin and related genes merely mark the cells progressing toward pluripotency, or functionally control cell fate transition into pluripotency, we sustained the level of actin cytoskeletal genes by expressing a constitutively active mutant MKL1 (caMKL1)^21^, or the control vector, in reprogrammable MEFs that also contain an Oct4:GFP knock-in reporter^25^ (Fig. 2a). The choice of expressing MKL1, instead of specific actin genes, is because MKL1/SRF concertedly upregulate many actin and related genes^19,20^, as is the case represented by the slow cycling cells (Supplementary Fig. 1b). A constitutively nuclear localized MKL1 was necessary because the wild type (WT) protein is inhibited by G-actin^21^, resulting in cytoplasmic localization (Fig. 1a, b) and ineffective upregulation of actin genes as reprogramming progresses. The MKL1 mRNA level yielded by ectopic expression was comparable to that of bulk MEFs (Fig. 2b). In the presence of Dox, both caMKL1-expressing and empty vector control MEFs gave rise to alkaline phosphatase (AP)+ colonies of ESC/iPSC-like morphology (Fig. 2c), indicating successful early reprogramming. However, unlike control iPSCs, caMKL1-expressing cells failed to activate the endogenous pluripotency reporter Oct4:GFP, even after prolonged Dox treatment (Fig. 2d, Supplementary Fig. 1e). They remained strictly dependent on exogenous reprogramming factors, as Dox withdrawal induced their rapid reversion to a fibroblastic morphology and loss of AP expression (Fig. 2e), suggesting that sustained MKL1 activity inhibits the activation of mature pluripotency.

Examination of reprogramming from additional somatic cell types, i.e. hematopoietic cells, confirmed the inhibitory effect by caMKL1 (Supplementary Fig. 2). Specifically, granulocyte and macrophage progenitors (GMPs), which reprogram efficiently as reported before^18,26,27^, naturally express lower actin-MKL1 pathway activity as compared to hematopoietic stem cells (defined as Lin-Kit + Sca + or LKS cells), which are not efficient in reprogramming despite being more primitive in their developmental stage (Supplementary Fig. 2a). caMKL1 expression (mCherry+) (Supplementary Fig. 2b) similarly and potently prevented the appearance of Oct4:GFP+ cells from reprogrammable GMPs (Supplementary Fig. 2c, d, e). Thus, MKL1 activity prevents activation of mature pluripotency from multiple somatic cell types.

To uncover the molecular consequences of MKL1 activity, we performed mRNA-seq and compared reprogrammable MEFs expressing control vector, or caMKL1, at early (Day 6) or late (Day 25) time points (Fig. 2f, g). As expected, the most significantly up-regulated gene ontology (GO) categories in caMKL1-expressing cells were actin-related biological pathways (Fig. 2f, g and Supplementary Fig. 3a, Supplementary Data 1), confirming MKL1 activity. To examine whether this altered gene expression program relates to the transcriptional activity of MKL1, we determined the binding of its transcriptional partner SRF by chromatin immuno-precipitation followed by sequencing (ChIP-seq), since a ChIP-grade antibody for MKL1 is not commercially available while SRF genome binding profile can be used to gauge MKL1 binding^28,29^. As expected, caMKL1 expression resulted in significantly stronger SRF binding to many cytoskeletal genes (Supplementary Fig. 3b, c, Supplementary Data 2), primarily to CArG motif sequences (Supplementary Fig. 3d). These data confirm that a main molecular consequence of caMKL1 expression is the elevation of many actin cytoskeletal components.

To position the cellular stage onto the molecular roadmap of pluripotency induction^30^, we compared the differentially expressed genes between day 25 control- and caMKL1-expressing cells to gene signatures of cells undergoing reprogramming (Supplementary Fig. 3e, f). Gene Set Enrichment Analysis (GSEA) showed that caMKL1-expressing cells failed to up-regulate many late pluripotency genes, such as *Esrrb* and endogenous *Sox2*, but sustained the expression of many others that should be downregulated during reprogramming (Supplementary Fig. 3e, f). Consistent with the gene expression data, ChIP-seq analyses of H3K4me3, H3K27ac and H3K27me3 confirmed the lack of active histone marks at the endogenous pluripotency loci in caMKL1-expressing cells (Supplementary Fig. 3g). While Oct4 bound to its bona fide target regions in the genome of control iPSCs, such as the *Oct4* enhancer, it was depleted from these regions in caMKL1-expressing cells (Fig. 2h, Supplementary Data 2). These caMKL1-expressing, AP+ cells appear to be in a unique state of pre-pluripotency since *Nanog* was expressed at a level comparable to control iPSCs (Supplementary Fig. 3h) and the corresponding histone marks at this locus were properly decorated (Supplementary Fig. 3g). These data demonstrate that MKL1 activity prevents the extensive activation of endogenous core pluripotency, yielding a cell state that is more advanced than previously reported reprograming intermediates^17,31,32^ but distinct from the Nanog+ F-class alternative pluripotency^33^.

**Fig. 3:**
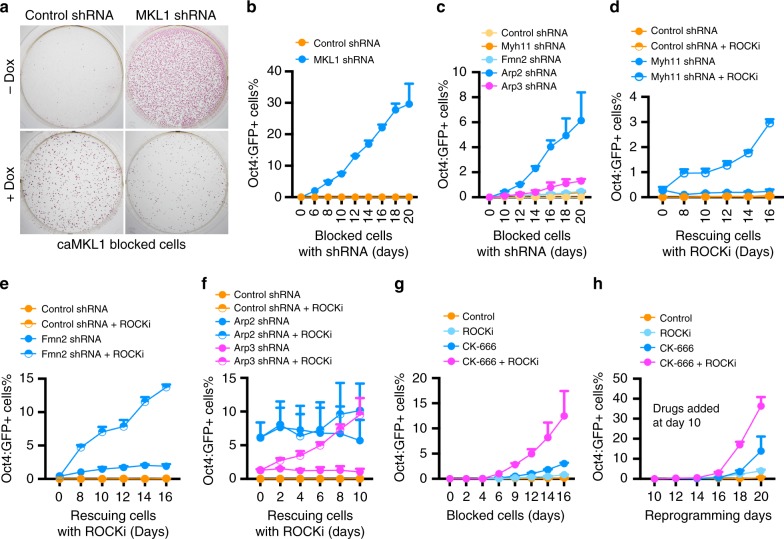
Actin polymerization is required for caMKL1-mediated block of mature pluripotency activation. **a** AP staining of caMKL1-expressing cells transduced with control or MKL1-targeting shRNA, cultured with or without Dox. Staining was performed 15 days after viral transduction. **b**–**f** Percentage of Oct4:GFP+ cells emerging from blocked cells following treatment of control or MKL1-targeting shRNAs (**b**); control or Myh11-, Fmn2-, Arp2- or Arp3-targeting shRNAs (**c**); control or Myh11-targeting shRNAs, with or without ROCKi (**d**); control or Fmn2-targeting shRNAs, with or without ROCKi (**e**); control or Arp2- or Arp3-targeting shRNAs, with or without ROCKi (**f**). **d**–**f** ROCKi was added at day 20 of shRNA treatment. **g** Percentage of Oct4:GFP+ cells emerging from blocked cells following treatment of ROCKi or CK-666, alone or in combination. **h** Percentage of Oct4:GFP+ cells emerging from reprogrammable MEFs in the presence of indicated drugs. Cells were treated with drugs starting at reprogramming day 10. All GFP+ percentages were determined by FACS at indicated time (days), following a gating strategy as shown in Supplementary Fig. 1. All data are representative of three independent experiments. Error bars denote standard deviation of triplicated samples (*n* = 3) in each experiment

The inhibition of mature pluripotency activation was not due to compromised cell proliferation and/or survival, because caMKL1-expressing MEFs gave rise to more AP+ colonies than controls (Fig. 2c), and these caMKL1-expressing AP+ colonies can be stably maintained in mESC culture conditions as long as Dox was added (Fig. 2c, d, e). Importantly, depleting MKL1 in these AP+ cells resulted in abundant mature iPSCs that could be maintained without Dox, whereas no mature colonies formed in control shRNA-treated cultures (Fig. 3a, b). Since these caMKL1-expressing cells remain in a stable pre-pluripotent state, which readily progressed toward mature pluripotency upon MKL1 inhibition, we refer to them as caMKL1-blocked cells.

### caMKL1-mediated block requires actin polymerization

The above analyses revealed two molecular consequences of MKL1 activity: elevated actin cytoskeletal gene expression and failed activation of mature pluripotency. Since high levels of actin cytoskeletal components favor polymerization, we tested whether actin polymerization leads to failure in mature pluripotency activation, first by modulating RhoA activity, a major driver of actin polymerization and actomyosin contractility^34,35^. As expected, RhoA activity promoted actin polymerization, as confirmed by the presence of strong phalloidin staining in cells expressing a constitutively active mutant RhoA^36^ (Supplementary Fig. 4a). This constitutively active mutant, but not wild-type or a dominant negative RhoA, decreased the number of Oct4:GFP+ colonies when expressed in reprogramming MEFs (Supplementary Fig. 4b). Thus, activating the actin cytoskeleton independent of caMKL1 expression could also compromise the activation of mature pluripotency, strongly suggesting that the elevated actin cytoskeleton likely mediates the MKL1 blocking effect of mature pluripotency.

To directly test whether MKL1-mediated blockade of mature pluripotency is afforded by the actin cytoskeleton, we depleted regulators of actin polymerization, namely Arp2/3 and Formin 2 (Fmn2)^7,37^, as well as one of the myosin heavy chains, Myh11 (Fig. 3c, Supplementary Fig. 5a). With varying efficiencies, knockdown of Myh11, Fmn2 or Arp2/3 in caMKL1-blocked cells all resulted in the emergence of Oct4:GFP+ cells and elevated the expression of endogenous mature pluripotency genes (Fig. 3c). Although the number of Oct4:GFP+ cells emerging after some of these shRNA treatments appeared moderate, these targeting shRNAs always yielded significantly more Oct4:GFP+ cells than control shRNA-treated cultures, which produced no Oct4:GFP+ cells at all. The effectiveness to restore mature pluripotency by interfering with these regulators of actin polymerization was lower than that of the positive control, when MKL1 itself was depleted (Fig. 3b). This is likely because caMKL1 upregulates many cytoskeletal genes, and inhibiting one at a time would likely not be as effective as inhibiting MKL1 itself. Indeed, the rescue effects of these shRNAs were greatly potentiated by an inhibitor (Y27632, ROCKi) of the Rho-associated protein kinase, which is known to activate actin polymerization (Fig. 3d, e, f and Supplementary Fig. 5b, c, d). Since Arp2/3 knockdown was most effective at releasing the blocked cells into mature pluripotency (Fig. 3c), we tested whether a small molecule inhibitor of Arp2/3 (CK-666) functions similarly. Indeed, CK-666 released the caMKL1-blocked cells into mature pluripotency, especially in the presence of ROCKi (Fig. 3g and Supplementary Fig. 5e). Together, these data demonstrate actin polymerization mediates MKL1-imposed restriction for mature pluripotency activation.

The potent blocking effect mediated by the actin cytoskeleton upon caMKL1 expression, and the release from the blockade upon their inhibition, prompted us to ask whether this actin polymerization-based mechanism antagonizes mature pluripotency induction during normal reprogramming in the absence of caMKL1. Indeed, CK-666 and ROCKi promoted the activation of endogenous pluripotency genes in normal reprogramming cultures when added at day 10 (Fig. 3h). These results support that the abundance and polymerization state of actins regulate nuclear reprogramming, and this regulatory effect is not limited to the blocked cell state created by caMKL1 expression.

### LINC complex contributes to the block of mature pluripotency

**Fig. 4:**
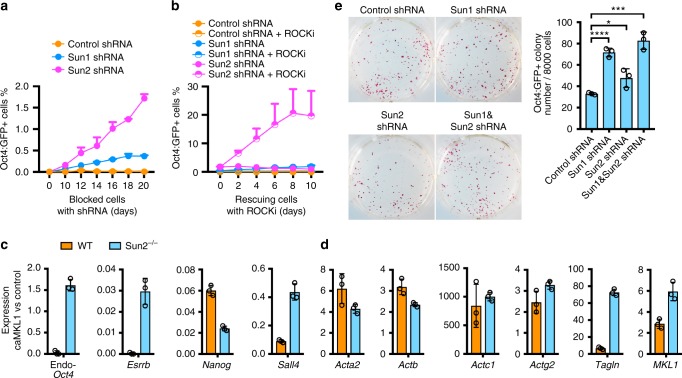
LINC complex contributes to the block of mature pluripotency activation. **a** Percentage of Oct4:GFP+ cells emerging from blocked cells following treatment of control, Sun1- or Sun2-targeting shRNAs. **b** The same experiments as in **a** were performed with or without ROCKi, which was added on day 20 of shRNA treatment. GFP+ percentages were determined by FACS at indicated time (days). **c**, **d** Realtime QPCR analyses of selected genes in control- or caMKL1-expressing cells, derived from reprogramming WT or *Sun2*^*−/−*^ cells on day 40. Each bar represents the ratio of the indicated gene expression levels in caMKL1- vs control-expressing cells. Endogenous pluripotency genes are shown in **c**; *MKL1* and its target genes are shown in **d**. Data are representative of three independent experiments. **e** AP stained cultures derived from reprogramming MEFs, while treated with control shRNA, Sun1- or Sun2-targeting shRNA, or both (left). The number of Oct4:GFP+ colonies were quantified on the right. *P < 0.05; ***P < 0.001; ****P < 0.0001. All data are representative of three independent experiments. Error bars denote standard deviation of triplicated samples (*n* = 3) in each experiment. Statistics were performed using two-tailed unpaired *t*-test

In addition to structurally supporting the cell, the actin cytoskeleton connects with the nucleus via a nuclear membrane-traversing molecular bridge, the LINC complex, which further connects to components of the nuclear interior via lamina-associated proteins^4,5^. To test whether this connection is involved in the blockade toward mature pluripotency, we inhibited components of the LINC complex. shRNAs against Sun1 or Sun2 (Supplementary Fig. 5a) each partially released the caMKL1-blocked cells into mature pluripotency (Fig. 4a), which was further enhanced by co-treatment with ROCKi (Fig. 4b). Strikingly, when Sun2 shRNA and ROCKi were added at the same time, the rescue efficiency reached a similar level as the positive control, MKL1 shRNA (compare Figs. 3b and 4b). These data suggest that the Sun2-containing LINC complexes together with polymerized actins are responsible for most of the blocking effects by caMKL1.

We further tested whether caMKL1-mediated block of mature pluripotency activation could be bypassed if the Sun2-containing LINC complex is genetically inactivated. As shown above, whereas caMKL1 expression completely prevented the activation of endogenous pluripotency genes, such as *Oct4* and *Esrrb*, in Sun2 wild type cells, these late pluripotency genes were activated in Sun2 KO cells^9^ even in the presence of MKL1 activity (Fig. 4c, d). Together, these data demonstrate that the LINC complex is required for MKL1 to restrict mature pluripotency activation.

To examine whether the LINC complex participates in the blockade of mature pluripotency in the absence of caMKL1, we inhibited either Sun1 or Sun2 during normal reprogramming. These treatment significantly increased the number of Oct4:GFP+ colonies (Fig. 4e). These data demonstrate that the LINC complex components are involved in restricting mature pluripotency activation. Importantly, these effects are not limited to the blocked cell state instituted by the expression of caMKL1. The involvement of the LINC complex components also suggests a potential mechanism how a hyperactive actin cytoskeleton could be responsible for the failure of the pluripotent cell fate.

### MKL1-actin pathway regulates nuclear state

**Fig. 5:**
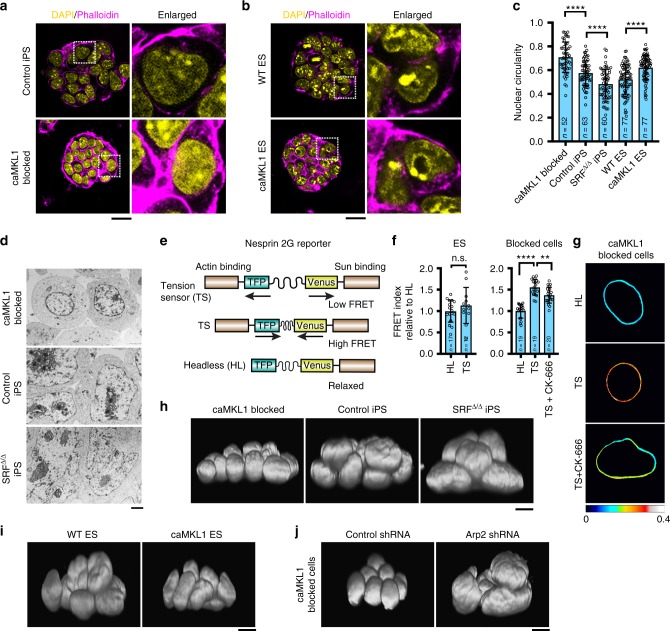
MKL1-actin pathway regulates nuclear volume. **a**, **b** DAPI and phalloidin staining in control iPS cells and caMKL1-blocked cells (**a**); WT and caMKL1-overexpressing ES cells (**b**). Rectangular boxes indicate regions shown at higher magnification on right. Scale bar: 20 μm. **c** Nuclear circularity of indicated cell types. The numbers of analyzed nuclei are as indicated. ****P < 0.0001. **d** Electron microscopy images showing nuclear morphology in caMKL1-blocked cells, control iPS cells and SRF^Δ/Δ^ iPS cells. Scale bar: 2 μm. **e** Schematics of the Nesprin-2G tension sensor (TS) and Headless control (HL). **f** FRET signals yielded by these constructs at the nuclear envelope were analyzed in blocked cells, or in mESCs as a control. CK-666 reduced the tension sensor FRET signals in the blocked cells. The numbers of analyzed nuclei are as indicated. n.s.: non-significant; **P < 0.01; ****P < 0.0001. **g** Representative heatmap images of FRET signals in caMKL1-blocked cells shown in **f**. **h**–**j** Representative 3D nuclear images reconstructed from DAPI stained confocal images of caMKL1-blocked cells, control iPS cells and SRF ^Δ/Δ^ iPS cells (**h**), WT and caMKL1-overexpressing ES cells (**i**), caMKL1-blocked cells treated with control or Arp2 shRNA (**j**). Scale bar: 10 μm. All data shown are representative of three independent experiments. Error bars denote standard deviation. Statistics were performed using two-tailed unpaired *t*-test

In searching for potential mechanism(s) how polymerized actins, together with the LINC complex, block mature pluripotency activation, we noticed that the nuclei of caMKL1-blocked cells were surrounded by abundant cytoplasmic F-actins, as indicated by the strong phalloidin signals (Fig. 5a and Supplementary Fig. 6a, b). Consistently, mESCs with ectopic caMKL1 also exhibited more abundant cytoplasmic F-actins surrounding the nuclei, while maintaining expression of pluripotency genes (Fig. 5b and Supplementary Fig. 6c, d, e). Of note, mESCs with high caMKL1 expression could not be established, indicating high caMKL1 activity might inhibit pluripotency.

The functional consequence of abundant F-actins toward the nucleus could be seen by analyzing isolated nuclei. Due to the nucleus’ viscoelastic nature^38,39^, isolated nuclei could temporarily retain their shape/size following their isolation but eventually swell and lose integrity. The ability to retain their integrity is reflected as the size of the isolated nuclei at defined time following isolation, with smaller nuclei indicating better retained integrity. Isolated nuclei from caMKL1-blocked cells always appeared smaller than those of control iPSCs (Supplementary Fig. 6b), and caMKL1-expressing mESCs similarly always yielded smaller nuclei (Supplementary Fig. 6d). Importantly, this caMKL1-induced difference was alleviated by actin de-polymerizing agent cytochalasin D (CD)^40^ in caMKL1-blocked cells as well as caMKL1-expressing mESCs (Supplementary Fig. 6b, d), indicating the involvement of actin cytoskeleton in generating the different nuclear states. In contrast, CD treatment had no discernable effects on control iPSC or WT mESC nuclei, consistent with their constitutively weak actin cytoskeleton (Fig. 1c, d). This assessment suggests that the abundant polymerized actins consequent to MKL1 activity cause qualitative differences in nuclear state.

We next analyzed the nucleus within intact cells. Indeed, the nuclei of the caMKL1-blocked cells appeared rounder than control iPSCs, as reflected by their higher nuclear circularity (Fig. 5c). This difference is not consequent to the different cellular state occupied by the blocked cells, because ectopic caMKL1 expression in mESCs also increased nuclear circularity (Fig. 5c). To further examine the relationship between MKL1/SRF activity and nuclear circularity, we generated SRF^Δ/Δ^ iPSCs from SRF^f/f^ cells^41^. Consistent with the phenotype of SRF-null mESCs^42^, SRF^Δ/Δ^ iPSCs maintained a pluripotent gene expression profile similar to SRF^f/f^ iPSCs (Supplementary Fig. 6f). SRF^Δ/Δ^ iPSCs had minimal F-actins with barely detectible phalloidin signals (Supplementary Fig. 6g), as expected from a disabled MKL1/SRF transcription program, while their nuclei were shaped much more irregularly (Fig. 5c). The difference in nuclear circularity was even more pronounced when the nuclei were examined with higher imaging resolution by electron microscopy (Fig. 5d). Overall, nuclear circularity increased with MKL1 activity, with the highest circularity detected in caMKL1-blocked cells and the lowest nuclear circularity in SRF^Δ/Δ^ iPSCs. These data further indicate that the nuclear state responds to MKL1/SRF activity.

Nuclear lamina is known to modulate nuclear morphology and chromatin states^43,44^. To determine whether nuclear lamina is altered in response to MKL1 activity, which in turn may mediate the blockade of mature pluripotency, we immunostained lamin A/C in the caMKL1-blocked cells. This revealed that lamin A/C protein was elevated in caMKL1-blocked cells (Supplementary Fig. 7a), and became detectible in mESCs when caMKL1 was overexpressed (Supplementary Fig. 7b). However, when we overexpressed lamin A or C in reprogramming MEFs directly, neither had any effect on reprogramming efficiency; similar numbers of Oct4:GFP+ colonies formed despite the overexpression of lamin A or C (Supplementary Fig. 7c–e). These data suggest that although caMKL1-expressing cells have elevated lamin A/C, the increased lamin A/C is likely one consequence of MKL1 activity, and has little direct antagonism to mature pluripotency activation. Nonetheless, these results further the observation that nuclear state responds to MKL1 activity. The abundant F-actins consequent to high MKL1 activity could profoundly change nuclear state, including altered nuclear lamina protein expression and nuclear morphology.

### MKL1-actin pathway regulates nuclear volume

To define the functional engagement between cytoplasmic actins and the nucleus, most likely via the LINC complex, we took advantage of a Nesprin-2G FRET sensor that consists of a pair of FRET-generating fluorophores, TFP and venus, connected by a linear-elastic spring linker^45^ (Fig. 5e and Supplementary Fig. 8a). The sensor is designed to have two binding domains from Nesprin-2G: one that binds actins on the outer nuclear envelope facing the cytoplasmic side, and the other that binds Sun proteins in the perinuclear space between the outer and inner nuclear membranes. The distance between the fluorophores is determined by strains on the two Nesprin domains, thus leading to altered FRET signals. A Headless (HL) construct lacking the actin-binding domain, which therefore exists in a relaxed configuration, serves as baseline FRET control. We found that caMKL1-blocked cells expressing the Tension Sensor (TS) displayed significantly higher FRET signals than those expressing the HL control (Fig. 5f, g), indicating that the FRET sensor was under compression in the caMKL1-blocked cells. Furthermore, the high FRET signal in caMKL1-blocked cells was reduced by treatment with the Arp2/3 inhibitor CK-666, indicating that the compression on the FRET sensor was at least partly mediated by polymerized actins (Fig. 5f,g). In contrast, no significant difference in FRET levels was seen between the TS and HL in mESCs (Fig. 5f), as expected from their constitutively weak actin cytoskeleton (Fig. 1c, d). These data support that the abundant actins present in caMKL1-blocked cells are functionally engaged with the nucleus via the LINC complex. The elevated Nesprin FRET signals in caMKL1-blocked cells suggest that the LINC complex is under compression in these cells.

It has been observed previously that when compressed, the nucleus reduces its size along the direction of compression^46^. The compressed Nesprin-2G FRET sensor prompted us to examine the relationship between MKL1-actin pathway activity and a potentially compressed nucleus, which may display decreased nuclear dimensions. To do this, we acquired and reconstructed 3D whole nuclei images from confocal image stacks of DAPI-stained nuclei. As shown in Fig. 5h and Supplementary Fig. 8b, caMKL1-blocked cells had the smallest nuclear volume (207 ± 58 μm^3^), while SRF^Δ/Δ^ iPSCs had the largest (553 ± 167 μm^3^), with control iPSCs in between (323 ± 176 μm^3^), indicating an inverse correlation between nuclear volume and MKL1 activity. In addition, caMKL1 expression in mESCs reduced their nuclear volume (Fig. 5i, from 352 ± 88 to 252 ± 44 μm^3^), suggesting MKL1 activity could directly reduce nuclear volume without causing significant changes in key pluripotency gene expression, likely involving nuclear compression. Further, treating caMKL1-blocked cells with Arp2-targeting shRNA restored nuclear volume (Fig. 5h, j, from 192 ± 53 to 701 ± 137 μm^3^). These data are in agreement with the functional rescue of caMKL1-blocked cells by Arp2 inhibition (Fig. 3c, f, g and Supplementary Fig. 5c, d, e). Mirroring the effects of ROCKi and CK-666 in inhibiting actin polymerization and promoting mature pluripotency, ROCKi and CK-666 also helped to increase nuclear volumes in late reprogramming (Supplementary Fig. 8c). Overall, these data are consistent with a model where nuclear volume is regulated by Arp2-dependent actin polymerization.

### MKL1 activity results in reduced chromatin accessibility

**Fig. 6:**
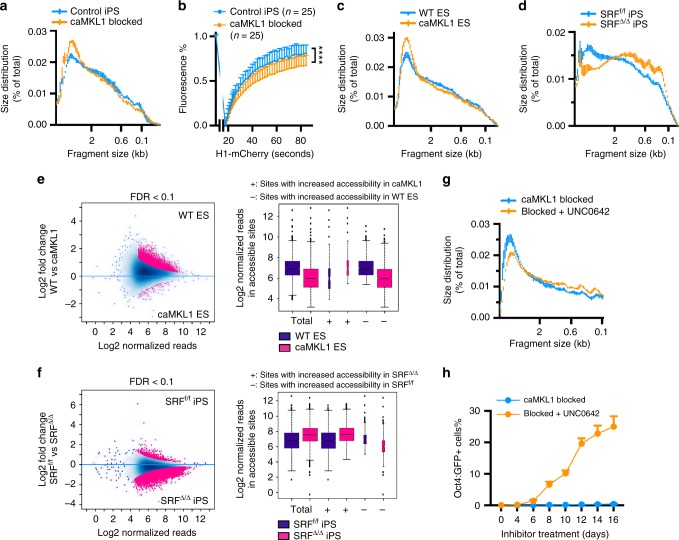
MKL1 activity results in reduced chromatin accessibility. **a** Quantification of the size distribution of DNase I digested DNA fragments from control iPS cells and caMKL1-blocked cells. *n* = 3 for each genotype. **b** FRAP analysis of the dynamic movement of histone H1^0^-mCherry in control iPS cell nuclei or caMKL1-blocked cell nuclei. ****P < 0.0001. *n* denotes the number of analyzed nuclei. Statistics were performed using two-tailed paired *t*-test. Error bars denote standard deviation. **c**, **d** Quantification of the size distribution of DNase I digested DNA fragments from WT and caMKL1-overexpressing ES cells (**c**) and SRF^f/f^ iPS cells and SRF^Δ/Δ^ iPS cells (**d**). *n* = 3 for each genotype. **e**, **f** MA plots of differential ATAC-seq peaks in WT vs caMKL1-expressing ES cells (**e**) and SRF^f/f^ vs SRF^Δ/Δ^ iPS cells (**f**). Pink dots indicate regions of significant differences in chromatin accessibility (left). The width of boxplots (right) is proportional to the number of regions of differential chromatin accessibility, showing overall lower accessibility in caMKL1-expressing ESCs (**e**), and overall increased accessibility in SRF^Δ/Δ^ iPS cells (**f**). The center line of boxplots indicate the median value; the edges of the boxplots are at the 25th and 75th percentile; the whiskers extend to 1.5 times the interquartile range past the box. Points that are outside whiskers are shown individually. **g** Quantification of the size distribution of DNase I digested DNA fragments from caMKL1-blocked cells treated with or without UNC0642. *n* = 3 for each genotype. **h** Percentage of Oct4:GFP+ cells emerging from blocked cells following treatment with UNC0642. GFP+ cells were determined by FACS. All data shown are representative of three independent experiments

A direct implication of a reduced nuclear volume as seen with caMKL1-blocked cells (Fig. 5h, Supplementary Fig. 8b) is a more compact chromatin organization, since the same amount of genome is confined to a smaller space in 3D. Such a model could effectively explain the failure of caMKL1-blocked cells in activating mature pluripotency, likely due to decreased chromatin accessibility. To test this possibility, we first performed DNase I digestion assay, which revealed overall reduced chromatin accessibility in caMKL1-blocked cells relative to control iPSCs (Fig. 6a). This reduction in chromatin accessibility is echoed and corroborated by reduced Oct4 chromatin binding as revealed by ChIP-seq, shown above in Fig. 2h. In addition, fluorescence recovery after photobleaching (FRAP) analysis of mCherry-tagged histone H1^0^ confirmed reduced chromatin dynamics in the blocked cells (Fig. 6b). The difference in chromatin openness is not consequent to differences in cellular states, as mESCs displayed a reduction in chromatin openness when caMKL1 was expressed (Fig. 6c). Furthermore, DNase I digestion assay revealed that SRF null iPSCs displayed increased chromatin accessibility beyond that of the control SRF^f/f^ iPSCs (Fig. 6d). Besides modulating MKL1/SRF, expression of the constitutively active RhoA mutant in mature iPSCs also decreased chromatin accessibility, while expression of the dominant negative RhoA mutant promoted it (Supplementary Fig. 9a). Taken together, the overall chromatin accessibility is modulated by the MKL1/SRF activity level as assessed by DNase I accessibility.

To examine the effects of MKL1/SRF activity on chromatin accessibility in more detail, we performed ATAC-seq^47^ comparing the genome-wide accessibility when MKL1/SRF activity is altered. To avoid measuring the difference in chromatin accessibility consequent to different cell state, we performed these comparisons in mESCs with or without caMKL1 expression, and in iPSCs with or without a functional SRF gene. Consistent with the DNase I digestion assay results (Fig. 6c, d), ATAC-seq analyses confirmed globally reduced chromatin accessibility in mESCs following caMKL1 expression (Fig. 6e and Supplementary Fig. 9b, Supplementary Data 3), including many Oct4 binding sites/regions (Supplementary Fig. 9c). Conversely, loss of SRF in iPSCs globally increased chromatin accessibility, beyond the level seen with wild type iPSCs (Fig. 6f). Together, these data demonstrate that MKL1 activity can result in a globally less accessible chromatin conformation.

To assess whether the reduced chromatin accessibility in caMKL1-blocked cells was responsible for their failed activation of mature pluripotency, we promoted chromatin accessibility by means independent of the MKL1-actin pathway. Specifically, we treated the caMKL1-blocked cells with UNC0642, an H3K9me2/3 methyltransferase inhibitor^48^. As expected, UNC0642 treatment increased chromatin accessibility in caMKL1-blocked cells (Fig. 6g). Importantly, this treatment alone resulted in the activation of endogenous pluripotency genes and led to the emergence of mature pluripotent stem cells that were independent of Dox (Fig. 6h and Supplementary Fig. 9d). These data suggest that compromised chromatin accessibility driven by caMKL1 could largely explain their failure in activating mature pluripotency. Alleviating this inhibition, either by inhibiting MKL1-actin pathway directly or by inhibiting the heterochromatin-forming enzymatic activity, restores chromatin accessibility and allows pluripotency activation.

## Discussion

In this report, we demonstrate that chromatin accessibility and mature pluripotency activation subject to negative modulation by the ubiquitously expressed transcriptional co-activator MKL1 and its transcriptional product actin cytoskeleton. High MKL1 activity elevates the actin cytoskeleton, which connects with the nucleus via the LINC complex. Sustained MKL1 activity results in reduced nuclear volume and globally reduced chromatin accessibility. A smaller nuclear space could presumably reduce the spatial distance separating distinct genomic loci, thereby exponentially increasing their contact probability^49^ or facilitating gene-silencing maintenance by PRC2^50^. Such a model would favor the stabilization of a given chromatin conformation, rather than its reorganization. This could potentially explain why the activation, but less so maintenance, of mature pluripotency is exquisitely sensitive to MKL1 activity.

Our work shifts the appreciation for the actin cytoskeleton-LINC system, from an architectural network simply providing nuclear structural support to an active contributor shaping the epigenome. Our data collectively demonstrate that key components of the mechano-transduction machinery could restrict nuclear dynamics on a global level, revealing that the nuclear process essential for cell fate reprogramming is modulated by mechanisms beyond the nuclear-localized epigenetic enzymes. The depiction of actin-LINC in restricting chromatin accessibility could explain how certain kinases of cytoskeletal dynamics^51^ or actin-targeting shRNAs promoted reprograming^52^ and offer mechanistic insights for how to integrate biophysical cues into cell fate engineering^53^. Our work demonstrates that the cytoskeletal changes accompanying reprogramming are not merely passive phenotypic changes, consequent to changes in chromatin remodeling. Instead, these changes could pose significant effects toward chromatin remodeling. By sustaining the function of a key molecular regulator for actin cytoskeleton, we uncovered and reenacted a molecular mechanism that is difficult to appreciate otherwise, when cell states are in constant transitions during cell fate reprogramming.

Since the MKL1-actin activity is ubiquitous, the proposed mechanism is likely relevant in other cellular contexts, including at the earlier stages of reprogramming or in ESC/iPSCs derived from other species. Intriguingly, one of the most distinct features between human and mouse pluripotent stem cells is their colony morphology, with human cells being flat monolayers of higher focal adhesion signaling^54^. It will be important to determine whether modulating actin cytoskeleton-LINC complex activity can lead to transitions between different pluripotent states in human ESC/iPSCs^55,56^, or in deriving bona fide pluripotent stem cells of certain challenging model species^57^. Our model might also provide new insights to biology beyond the regulation of pluripotency, such as in the emergence of aberrant cell states underlying the various fibrotic conditions^58,59^.

## Methods

### Mice and cells

All mouse work was approved by the Institutional Animal Care and Use Committee of Yale University. The reprogrammable mouse (*R26*^*rtTA*^*;Col1a1*^*4F2A*^)^60^ (stock# 011004) was purchased from the Jackson Laboratory. The reprogrammable mice with reporter (*R26*^*rtTA*^*;Col1a1*^*4F2A*^*;Oct4*^*GFP*^) were derived by crossing reprogrammable mice with *Oct4:GFP* mice^26,61^. *SRF*^*f/f*^*R26*^*rtTA*^*TetO:Cre* mouse line was derived by crossing the *SRF*^*f/f*^ (stock# 006658) with tetO:Cre (stock# 006234). Sun2 knockout mouse has been described before^6,9^. MEFs with different genetic background were all derived from E13.5 embryos. Granulocyte-Monocyte progenitors (GMPs) were collected from the bone marrow of mice with corresponding genotypes with the immune-phenotype of Lin-Sca-Kit + CD34 + CD16/32 + ^18,61^. Embryonic stem cells (ESCs) were derived from wild-type C57BL/6 E3.5 embryos.

### iPS cell induction

For reprogrammable MEFs, reprogramming was induced by adding Doxycycline (Dox) to the ESC culture medium with a final concentration of 2 μg/mL. For overexpression or knockdown experiments during reprogramming, viruses were generally transduced or co-transduced one day before changing the medium with or without Dox. The day when Dox is on is day 0. For all MEF reprogramming experiments, medium was changed every other day. On day 4, reprogramming MEFs were trypsinized and seeded onto feeder layer with the number of cells indicated. Unless otherwise indicated, AP staining was generally performed on day 10 or day 12, depending on the size of the colonies. For reprogramming involving more than 12 days, reprogramming cells were passaged and replated onto feeders at indicated time points. In selected cases (Sun1/2 knockdown, lamin A/C overexpression) where Oct4:GFP+ colonies could appear without cell passaging, vitamin C was added into the culture medium starting on day 4. For GMPs, reprogramming cells were seeded onto feeder layer and monitored for iPS colony formation without changing medium during the entire process.

### Blocked cell maintenance and rescue

Blocked cells were cultured on feeder layers with ESC medium in the presence of 2 μg/mL Dox. For rescue experiments, cells were seeded onto gelatin-coated plates one day before viral transduction. Cells were incubated with viral medium supplemented with 5 μg/mL polybrene (Millipore), and centrifuged at 2500 rpm, RT for 90 min. Cells were then incubated in the medium containing viral particles in 37 °C incubator for another 24 h. After that, cells were trypsinized and replated onto feeder layer, cultured in ESC medium with Dox to monitor rescuing activity. For rescue experiments with small molecules, drugs were added to the medium, and cells were passaged every other day. ROCKi (Y-27632, CalBiochem, 688000) was used at 10 μM, CK-666 (Sigma, SML-0006) was used at 50 μM, and UNC0642 (Sigma, SML-1037) was used at 1 μM.

### Plasmids

MKL1-GFP construct was generated by PCR amplifying MKL1 from pcDNA5/TO-MKL1^62^, replaced the IRES sequence and fused with the EGFP in MIG-R1. Constitutively active MKL1 (caMKL1) was generated by deleting the first 240 nucleotides (NM 001082536) encoding the RPEL domain and adding the SV40 NLS before the remaining sequence. RhoA constructs were purchased from Addgene (12965, 12967, 12968)^36^ and subcloned into the pMSCV-IRES-mCherry FP backbone (52114). LMNA/C construct was purchased from addgene (17662)^63^ and subcloned into the pMSCV-IRES-mCherry FP backbone, or fused with a mCherry at the N-terminus and subcloned into the pMSCV backbone. H1f0 was subcloned into the pMSCV backbone^15^, with a mCherry fusion. Nesprin-2G headless and tension sensor was obtained from Addgene^45^. The coding sequence of headless and tension sensor were subcloned into the pFUW lentiviral backbones under the control of the TetO inducible promoter. MKL1 shRNA construct was generated by inserting the short hairpin sequence into the lentiviral backbone psi-LVRU6MP (GeneCopia). Myh11, Fmn2, Arp2, Arp3, Sun1, and Sun2 shRNA constructs were generated by inserting the short hairpin sequence into the lentiviral backbone pLKO.1-blast. Each gene has 2–3 shRNA constructs, while only one is shown. Sequence information is provided in Supplementary Table 1.

### Chromatin immunoprecipitation (ChIP)

Detailed method is provided in Supplementary Methods. For immunoprecipitation, Oct4 antibody (Cell Signaling, 5677S) and SRF antibody (Active Motif, 61385, Santa Cruz, sc-335) were used at 6 μg/ChIP sample, H3K4me3 antibody (Millipore, 07-473), H3K27ac antibody (Abcam, Ab4729), and H3K27me3 antibody (Millipore, 07-449) were used at 1 μg/ChIP sample.

### RNA-seq and data analysis

RNA-seq libraries were prepared with TruSeq Stranded mRNA Library Prep Kit (Illumina, RS-122-2101) following the manufacturer’s instructions. High-throughput sequencing was performed with the Illumina HiSeq 2000 Sequencing System. For data analysis, the RNA-seq reads were mapped to mouse genome (mm10) with Bowtie2 in local mode, which allows the reads spanning the exon-exon junctions to get mapped to one of the two exons (whichever gives the higher mapping score) independent of the transcriptome annotation. Differentially expressed genes were identified by DESeq2 (v1.16.0) followed by cutting off with FDR-adjusted *P* value < 0.05 and fold changes > 2. MA plot of differentially expressed genes was also done with the R software. Gene Set Enrichment Analysis (GSEA) was performed with the GSEA software.

Reference gene sets were obtained from Polo et al.^30^. GO analysis of differentially expressed genes was performed with DAVID (https://david.ncifcrf.gov).

### ChIP-seq and data analysis

ChIPed DNA was tested for quality with BioAnalyzer (Agilent) before library preparation. Libraries were constructed with ThruPLEX^®^ DNA-seq Kit (Rubicon Genomics) following the manufacturer’s instructions. High-throughput sequencing was performed with the Illumina HiSeq 2000 Sequencing System. ChIP-seq reads were aligned to the mouse genome mm10 using Bowtie2 (v2.2.6) with default settings. For histone modifications, the resulting BAM files were filtered to remove the reads that have the same sequence, and converted to reads coverage files using the BamCoverage in DeepTools (v2.5.0). Reads coverage files were normalized as RPKM (Reads Per Kilobase of transcript per Million mapped reads) and visualized in IGV (v2.3). Heatmap graphs were generated with DeepTools (v2.5.0). For transcription factors, filtered BAM files were used for peak calling and differential peak analysis using MACS2 (v2.1.1). Differential peaks were annotated with PAVIS (https://manticore.niehs.nih.gov/pavis2/). GO analysis of differential peaks was performed with DAVID (https://david.ncifcrf.gov). Motif analysis was performed by HOMER software.

### Nuclei preparation, DNase I digestion, and quantification

For nuclei preparation, live cells were suspended in RSB buffer (10 mM Tris-Cl, pH 7.4, 10 mM NaCl, 3 mM MgCl_2_), and lysed by adding equal volume of RSB buffer supplemented with 0.2% IGEPAL^®^ CA-630 (Sigma, I8896). Nuclei were spun down at 500 g, 4 °C, washed once with ice-cold DPBS, and resuspended in 1× DNase I buffer in a concentration of 5 million nuclei/1 mL. Generally, aliquots of 100 k to 250 k nuclei were used for DNase I digestion (Roche, 04716728001). Nuclei were digested with 0.1–0.4 U/μL of DNase I at 37 °C for 5 min, and stopped with equal volume of stop buffer (50 mM Tris-HCl, pH 8.0, 100 mM NaCl, 100 mM EDTA, pH 8.0, 0.1% SDS). RNA was removed by adding RNase A (20 μg/mL) and incubated at 37 °C for 30 min. Protein was subsequently digested by adding Proteinase K (20 μg/mL) and incubated at 55 °C overnight. DNA was extracted by Phenol-Chloroform extraction. Reconstituted DNA was used for real-time PCR analysis or agarose gel running. For quantification of the agarose gel image, the intensity of DNA signals in each lane was quantified by GelAnalyzer2010a. Plots were generated by calculating *I* = (*I*_n_−*I*_b_)/*I*_total_, where *I* is intensity, n is the location on the gel, b is background, and *I*_total_ is sum of *I*_n_-*I*_b_.

### ATAC-seq and analysis

Detailed method for ATAC-seq is provided in Supplemetary Methods. For ATAC-seq analysis, sequence reads were trimmed using the trimmomatic software to remove nextera adapters and low quality bases at the beginning and end of the reads in paired end mode^64^. Reads were then aligned to the mouse mm10 genome sequence using the bwa mem mapper (arXiv:1303.3997 [q-bio.GN]). Reads were sorted and duplicates were marked using the Picard software (http://broadinstitute.github.io/picard/). Sequence reads that aligned with poor mapping quality to problematic black list regions or mitochondrial regions were removed. Peaks were called on reads with reads with insert size smaller than 250 bases using the MACS2 peak caller using the paired end mode -f BAMPE option. Differentially bound peaks were identified using the DiffBind R package utilizing the Deseq2 method with parameters fragmentSize = 300 and bFullLibrarySize = TRUE (http://bioconductor.org/packages/release/bioc/html/DiffBind.html). Peaks were normalized by the total number of reads from all the samples.

### Antibody dilution for immunostaining

Lamin A/C antibody (Santa Cruz, sc376248) was used at 1:200 at 4 °C overnight.

### Phalloidin staining, DAPI staining and nuclei analysis

For Phalloidin staining, Alexa Flour 660-conjugated Phalloidin (ThermoFisher, A22285) was used at RT for 30 min. DAPI (ThermoFisher, D1306) staining was performed at RT for 5 min. Images were acquired by confocal microscopy (Leica SP5). Nuclear area and nuclear intensity and nuclear circularity were measured with DAPI stained nuclei confocal images in Image J, where circularity is defined as 4 × π (area perimeter^−2^). Fifty-five to eighty nuclei were randomly selected for each genotype. For 3D reconstruction of the nuclei, z-stack images were acquired on Leica SP5 confocal microscopy with z depth 0.25 μM. Images were stacked with the Leica software LAS X.

### Nuclear volume measurement

Nuclear volume was measured with the Imaris program. z-stack images were loaded to Imaris and converted to Imaris 3D image files (.ims). The surface detail was standardized at 0.002 mm for all images analyzed. The Surface Detail tool was used to control the surface of the nuclear images, and he Background Subtraction tool was used to identify high confidence nuclei. The Threshold (Background Subtraction) was kept at the computer autogenerated average and the Split Touching Objects (Region Growing) option was enabled. The Seed Point Diameter was used to ensure individual nuclei could be analyzed as distinct objects, since many nuclei partially overlap (especially with the Arp2 shRNA or SRF null cells). Only the nuclei that were adequately separated from each other were recorded. The nuclei that overlapped with each other and could not be segmented confidently were discarded. When the surface was finalized, the tool Statistics was used to with the Detailed option and Specific Values tab selected. The Volume tab was used to generate the nuclear volumes.

### FRAP assay

Blocked cells or control iPS cells were transduced with viruses expressing the H1f0-mCherry fusion protein. Images were acquired on a Leica SP5 confocal microscope. All experiments were done at 37 °C supplemented with 5% CO_2_. For the FRAP experiments a pre-bleach image was acquired by averaging three consecutive images. Then a single spot of 2 μm × 2 μm region of interest (ROI_1_) on the nuclei was bleached with the UV and 531 nm laser pulses at the same time for 25 times lasting approximately 20 s, at 100% power of both lasers without scanning. For image collection, fluorescence was excited at 531 nm with 20% power, and collected at 565–670 nm. 10 single section images were collected at 0.78 s intervals, followed by 30 single section images collected at 2 s intervals. An ROI signal on the fluorescent nuclei without bleaching (ROI_2_) and an ROI signal of the background (ROI_3_) were collected for normalization. Plots were generated by calculating *I* = [*I*_t_ − *I*_4_]/(*I*_ave_ − *I*_4_), where *I*_t_ = (*I*_t.ROI1_ − *I*_t.ROI3_)/(*I*_t.ROI2_ − *I*_t.ROI3_), *I*_ave_ is the average *I*_t_ of the three pre-bleach images, and *I*_4_ is the first *I*_t_ post-bleach.

### FRET analysis

High resolution live FRET imaging was performed on Nikon Eclipse Ti widefiled microscope equipped with a cooled charged-coupled device Cool SNAP HQ2 camera, using a ×100, 1.49 NA oil objective at 37 °C. Images were acquired using Micromanager software. Three sequential images with 500 ms exposure time were acquired with the following filter combinations: donor (Teal) channel with 460/20 (excitation filter-ex), T455lp (dichroic mirror-di) and 500/22 (emission filter-em); FRET channel with 460/20 (ex), T455lp (di) and 535/30 (em); and acceptor (Venus) channel with 492/18 (ex), T515lp (di) and 535/30 (em) filter combinations. All filters and dichroic were purchased from Chroma Technology. For data analysis, donor leakage was determined from NIH3T3 cells transiently transfected with Vinculin-Teal, whereas acceptor cross excitation was obtained from Vinculin-Venus transfected cells. For all the calculations, respective background subtraction, illumination gradient, and pixel shift correction were performed followed by three-point smoothening. The slope of pixel-wise donor or acceptor channel intensity versus FRET channel intensity gives leakage (*x*) or cross-excitation (*y*) fraction, respectively. FRET map and pixel-wise FRET index for the sensors were determined from

$${\mathrm{FRET}}\,{\mathrm{index}} = \, \left[ {\mathrm{FRET}}\,{\mathrm{channel}} - x\left( {{\mathrm{Donor}}\,{\mathrm{channel}}} \right)\right. \\ \left. - y\left( {{\mathrm{Acceptor}}\,{\mathrm{channel}}} \right)\right]/\left[ {{\mathrm{Acceptor}}\,{\mathrm{channel}}} \right].$$

Mask for each cell was drawn manually based on the intensity around the nucleus. Mean FRET index per cell was calculated for each region within the mask^65^.

### Reporting Summary

Further information on experimental design is available in the Nature Research Reporting Summary linked to this article.

## Supplementary information

Supplementary Information

Reporting Summary

Description of Additional Supplementary Files

Supplementary Data 1

Supplementary Data 2

Supplementary Data 3

## Data Availability

Datasets from RNA-seq, ChIP-seq and ATAC-seq are available under the accession number GSE120399 in GEO [https://www.ncbi.nlm.nih.gov/geo/query/acc.cgi?acc=GSE120399]. All relevant data are available upon reasonable request.
