# Supplementary material for: MKL1-actin pathway restricts chromatin accessibility and prevents mature pluripotency activation
Source: Nat Commun. 2019 Apr 12;10:1695. doi: 10.1038/s41467-019-09636-6 (PMC6461646; doi:10.1038/s41467-019-09636-6)
Supplement: Supplementary file 1 — Supplementary Information [file 41467_2019_9636_MOESM1_ESM.pdf]

Supplementary Information

MKL1-actin pathway restricts chromatin accessibility and prevents mature pluripotency activation

Hu et al.

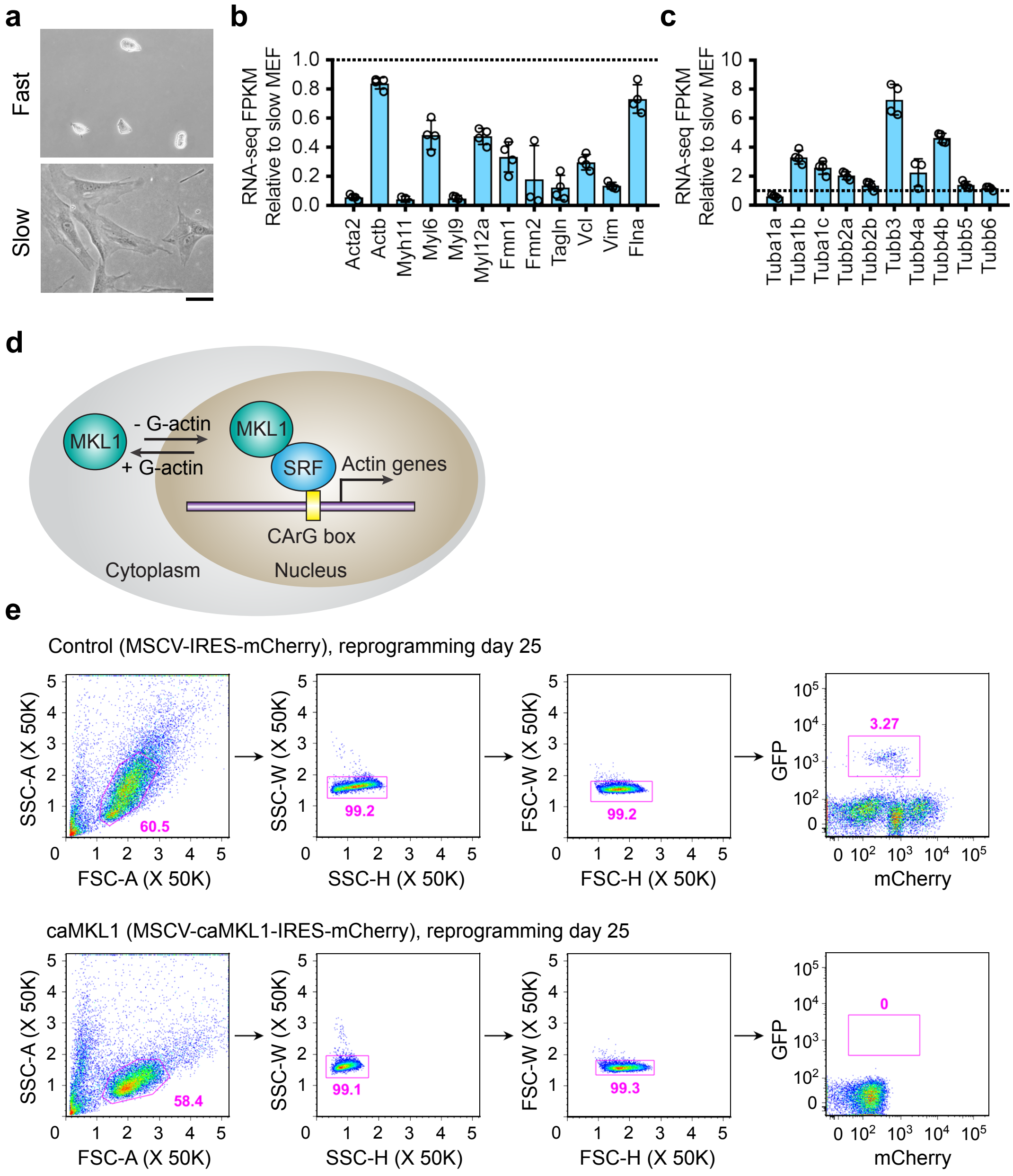

Supplementary Figure 1. Reduced activity in the actin-MKL1/SRF pathway accompanies reprogramming.
a, Representative cell size/morphology of fast and slow cycling cells sorted from reprogramming MEF cultures on day 6.
b, **c**, Relative mRNA levels of actin related genes (**b**) and tubulin related genes (**c**) in fast and slow cycling cells sorted from reprogramming MEF cultures on day 6. The number of reads for indicated genes in fast cycling cells were normalized to that of slow cycling cells, which were arbitrarily set to 1 (dashed line). Data were replotted from those described in Guo et. al., 2014. Error bar denotes standard deviation of four biological replicates within the dataset.
d, Schematics of the actin-MKL1/SRF signaling pathway. MKL1 nuclear translocation is regulated by its binding to monomeric actins (G-actin), which retains MKL1 in the cytoplasm. When cytoplasmic G-actin concentration drops, MKL1 is released from G-actin and accumulates in the nucleus to partner with SRF. The MKL1/SRF complex binds to the CArG box sequences in promoters to activate target gene expression, such as actin cytoskeletal genes.
e, Representative FACS gating strategy of reprogramming cells at day 25, overexpressing control or caMKL1. Cells were sequentially gated based on forward scatter (FSC), side scatter (SSC), and GFP intensity.

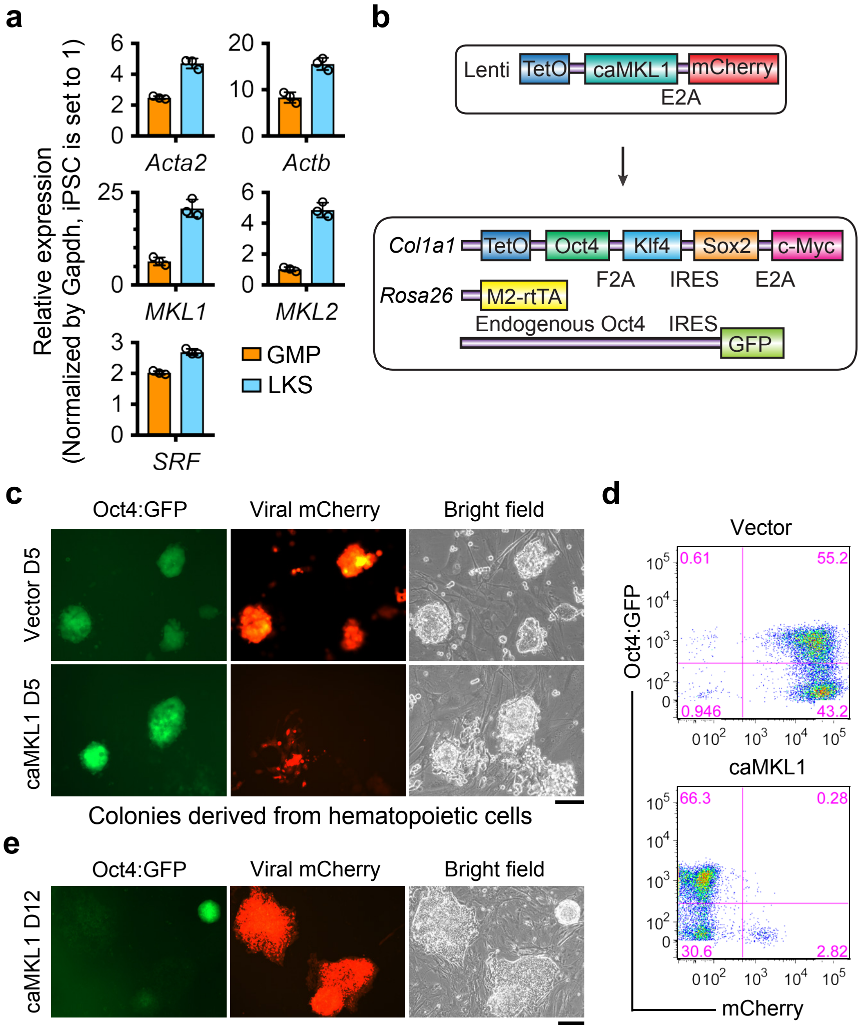

Supplementary Figure 2. caMKL1 inhibits hematopoietic progenitor reprogramming.

a, Realtime QPCR analyses of selected actin-MKL1/SRF pathway genes in granulocyte and macrophage progenitors (GMP) and Lin−Kit+Sca+ cells (LKS). Error bars denote standard deviation of three biologically independent samples.

b, Schematics of the experimental design to test caMKL1 effect on GMP reprogramming. Reprogrammable GMPs were transduced with control or caMKL1 lentiviral construct and induced for reprogramming by adding Dox.

c, Representative images of colonies derived from reprogrammable GMPs after viral transduction of control- or caMKL1-expressing vector depicted in **b**. Colonies were imaged at day 5. Scale bar: 100 µm.

d, Representative FACS plots of reprogramming cultures in **c**. Note the large number of mCherry+/Oct4:GFP + cells present in the control culture, and the absence of the double positive population in caMKL1-transduced culture. caMKL1 and Oct4:GFP expression are largely mutually exclusive.

e, The few Oct4:GFP+/mCherry+ cells present in the caMKL1-trasnduced reprogramming GMP culture (shown in d bottom) were FACS sorted, and continued to reprogram till day 12 and imaged. caMKL1-mCherry and Oct4:GFP are mutually exclusive. Scale bar: 100 µm.

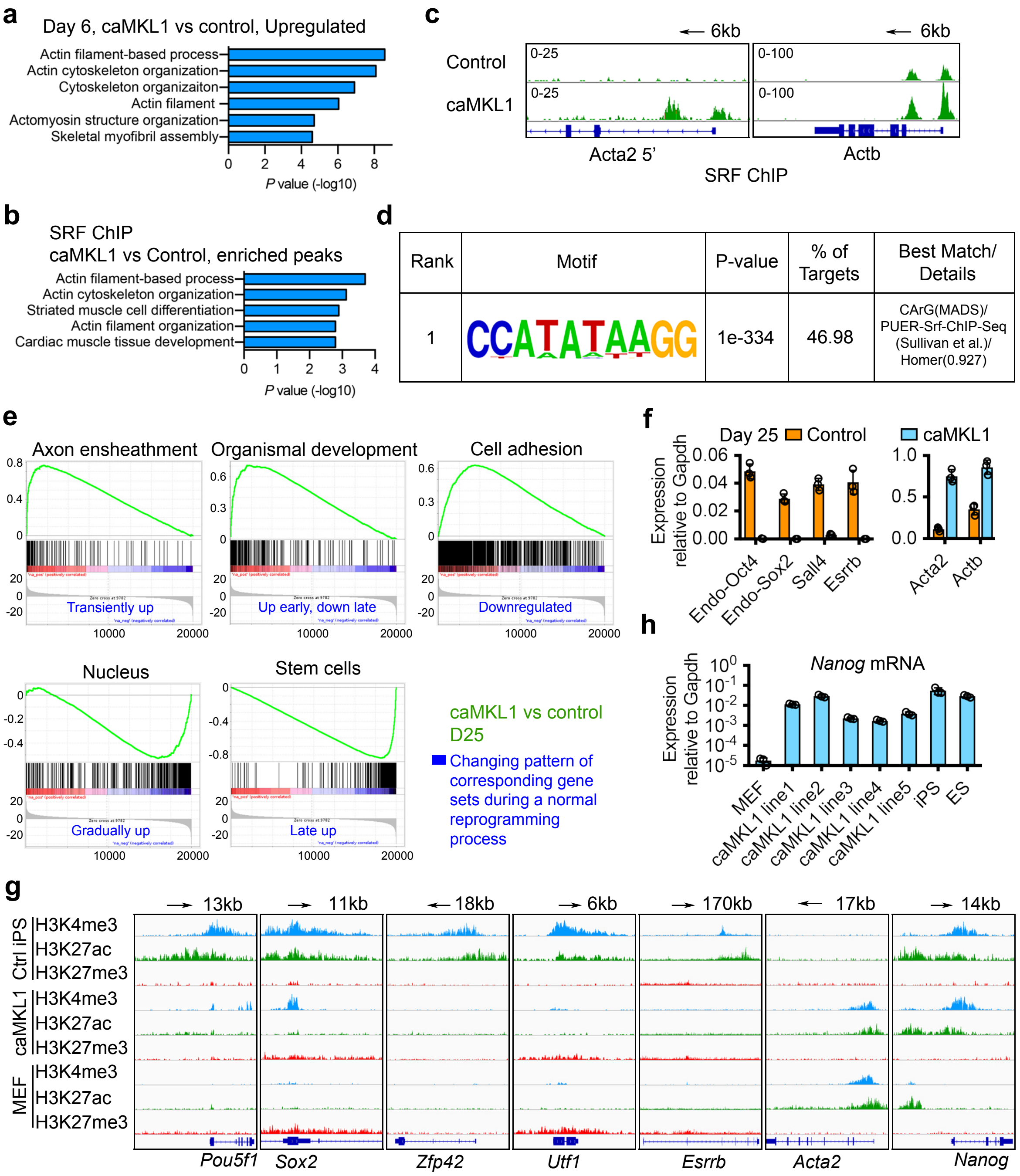

a
Day 6, caMKL1 vs control, Upregulated
Actin filament-based process
Actin cytoskeleton organization
Cytoskeleton organizaiton
Actin filament
Actomyosin structure organization
Skeletal myofibril assembly
0 2 4 6 8
P value (-log10)
b
SRF ChIP
caMKL1 vs Control, enriched peaks
Actin filament-based process
Actin cytoskeleton organization
Striated muscle cell differentiation
Actin filament organization
Cardiac muscle tissue development
0 1 2 3 4
P value (-log10)
c
6kb
6kb
Control
caMKL1
0-25
0-25
0-100
0-100
Acta2 5'
Actb
SRF ChIP
d
Rank
Motif
P-value
% of Targets
Best Match/ Details
1
CCATATAAGG
1e-334
46.98
CArG(MADS)/ PUER-Srf-ChIP-Seq (Sullivan et al.)/ Homer(0.927)
e
Axon ensheathment
Organismal development
Cell adhesion
Transiently up
Up early, down late
Downregulated
Nucleus
Stem cells
Gradually up
Late up
10000
20000
caMKL1 vs control D25
Changing pattern of corresponding gene sets during a normal reprogramming process
f
Day 25
Control
caMKL1
Expression relative to Gapdh
Endo-Oct4
Endo-Sox2
Sall4
Esrrb
Acta2
Actb
g
13kb
11kb
18kb
6kb
170kb
17kb
14kb
Ctrl iPS
caMKL1
MEF
H3K4me3
H3K27ac
H3K27me3
Pou5f1
Sox2
Zfp42
Utf1
Esrrb
Acta2
Nanog
h
Nanog mRNA
Expression relative to Gapdh
MEF
caMKL1 line1
caMKL1 line2
caMKL1 line3
caMKL1 line4
caMKL1 line5
iPS
ES

Supplementary Figure 3. caMKL1 overexpression induces excessive actin related gene expression during reprogramming and results in a Nanog+ cell state that is not pluripotent.

a, mRNA-seq analysis identifying differentially expressed genes between caMKL1-overexpressing cells and control vector-expressing cells on reprogramming day 6. GO analysis reveals cytoskeletal genes are collectively up-regulated in caMKL1-overexpressing cells.

b, SRF ChIP-seq analysis comparing SRF binding to chromatin in caMKL1-overexpressing cells and control vector-expressing cells on reprogramming day 25. Genes display increased SRF binding in caMKL1-overexpressing cells largely belong to the actin cytoskeletal genes, as revealed by GO analysis.

c, Genome browser screenshots of two representative SRF binding peaks (*Acta2* 5' and *Actb*) in the dataset described in **b**.

d, Motif analysis (HOMER) of the differential SRF-bound peaks in the dataset described in **b**.

e, Gene set enrichment analysis (GSEA) of differentially expressed genes between caMKL1-overexpressing cells and control vector-expressing cells on reprogramming day 25.

f, Realtime QPCR verification of endogenous pluripotency gene expression in control and caMKL1-overexpressing cells on reprogramming day 25. n = 3. Data are mean and s.d. Data are representative data of three independent experiments.

g, Representative genome browser screenshots of H3K4me3, H3K27ac and H3K27me3 ChIP-seq analysis performed with MEFs, caMKL1-overexpressing cells (day 25), and control iPS cells (day 25). Representative genomic loci of several differentially expressed genes display differential decoration by these histone marks. caMKL1-overexpressing cells failed to gain active marks at pluripotency genes (*Pou5f1*, *Sox2*, *Zfp42*, *Utf1* and *Esrrb*), retained some of the fibroblast identity marks (*Acta2*), but have gained active marks in the *Nanog* locus.

h, Realtime QPCR analyses of *Nanog* expression in MEFs, five independently derived caMKL1-overexpressing cell lines, control iPS cells and ES cells. Error bar denotes standard deviation of triplicate samples.

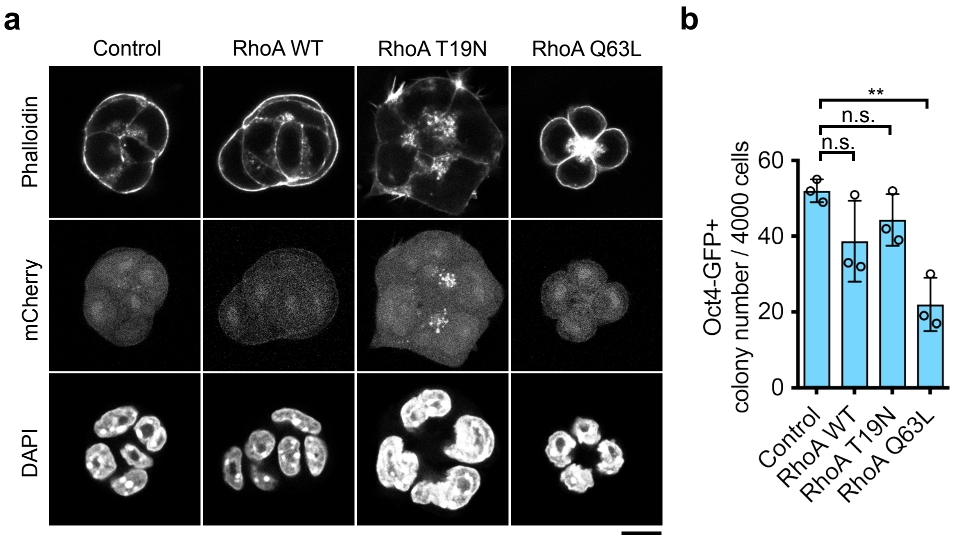

Supplementary Figure 4. RhoA activity promotes F-actin formation and blocks reprogramming.
a, Representative phalloidin-stained iPSC colonies ectopically expressing control vector, RhoA WT, RhoA T19N (dominant negative) or RhoA Q63L (constitutively active). mCherry is co-expressed from the same vector and indicates successfully transduced cells. Nucleus were stained with DAPI. Scale bar: 20 μm.
b, Quantification of Oct4:GFP+ colonies generated from reprogrammable MEFs transduced to express control, RhoA WT, RhoA T19N (dominant negative) or RhoA Q63L (constitutively active). n.s.: non-significant; **: $P < 0.01$. n = 3. Data are mean and s.d. Data shown are representative of three independent experiments. Statistics are performed using two-tailed unpaired *t*-test.

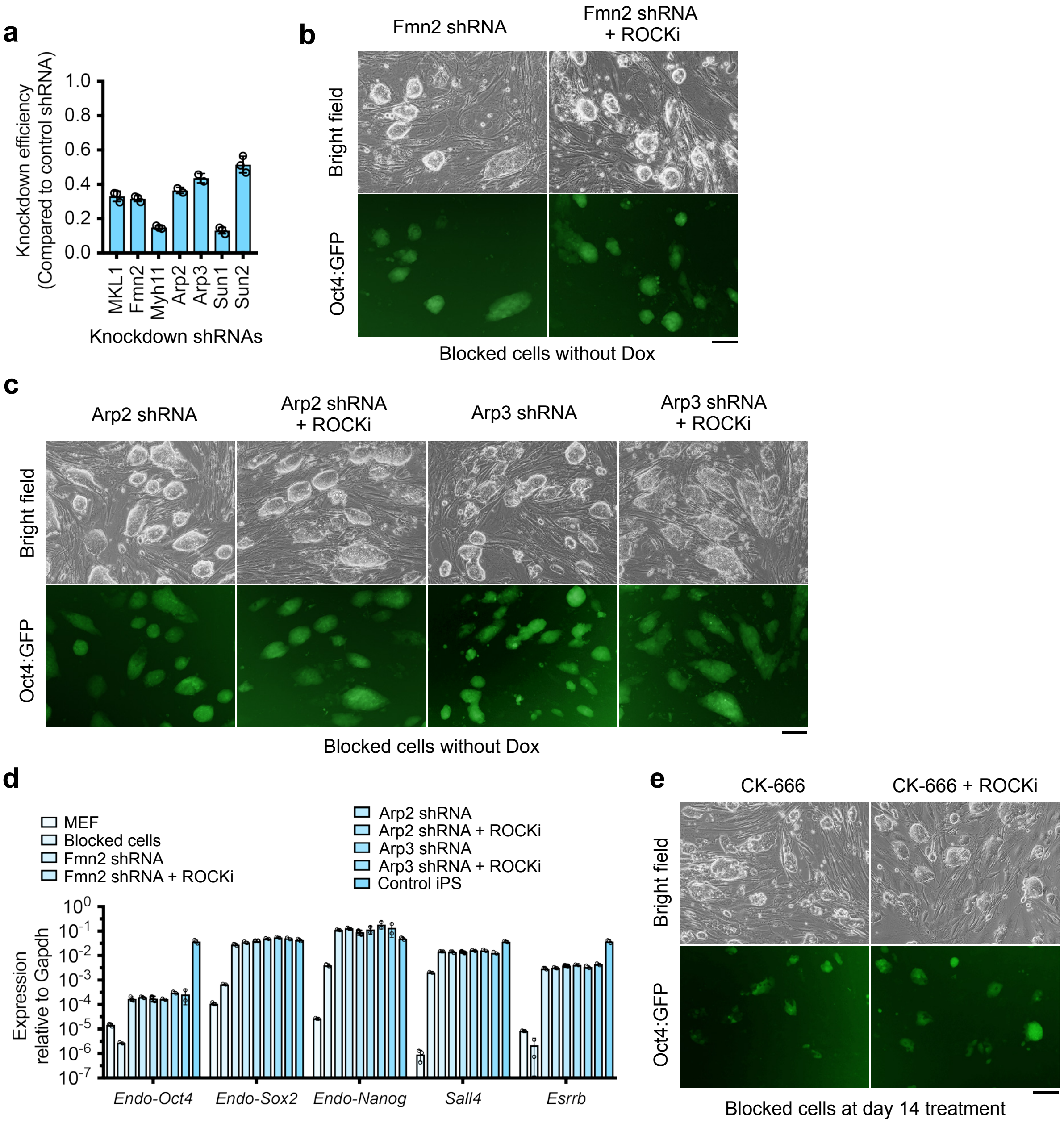
a
Knockdown efficiency (Compared to control shRNA)
1.0
0.8
0.6
0.4
0.2
0.0
MKL1
Fmn2
Myh11
Arp2
Arp3
Sun1
Sun2
Knockdown shRNAs
b
Fmn2 shRNA
Fmn2 shRNA + ROCKi
Bright field
Oct4:GFP
Blocked cells without Dox
c
Arp2 shRNA
Arp2 shRNA + ROCKi
Arp3 shRNA
Arp3 shRNA + ROCKi
Bright field
Oct4:GFP
Blocked cells without Dox
d
MEF
Blocked cells
Fmn2 shRNA
Fmn2 shRNA + ROCKi
Arp2 shRNA
Arp2 shRNA + ROCKi
Arp3 shRNA
Arp3 shRNA + ROCKi
Control iPS
Expression relative to Gapdh
10^0
10^{-1}
10^{-2}
10^{-3}
10^{-4}
10^{-5}
10^{-6}
10^{-7}
Endo-Oct4
Endo-Sox2
Endo-Nanog
Sall4
Esrrb
e
CK-666
CK-666 + ROCKi
Bright field
Oct4:GFP
Blocked cells at day 14 treatment

Supplementary Figure 5. Inhibition of actin polymerization rescues blocked cells toward mature pluripotency.

a, Realtime QPCR analysis of knockdown efficiency for the indicated genes. The mRNA level for each gene present in control shRNA-treated cells were set as 1. n = 3. Data are mean and s.d. Representative data from three independent experiments are shown.

b, **c**, Representative images of emerging Oct4:GFP+ colonies from blocked cells following shRNA treatment against Fmn2 (**b**) , Arp2 or Arp3 (**c**) , or together with ROCKi. Cells are no longer dependent on Dox (exogenous OKSM). Scale bar: 100 μm.

d, Realtime QPCR analyses of selected pluripotency genes in blocked cells following treatment of shRNAs against Fmn2, Arp2 or Arp3, with or without ROCKi. The levels of these genes in MEFs, caMKL1-blocked cells and control iPS cells are plotted as controls. n = 3. Data are mean and s.d. Representative data from three independent experiments are shown.

e, Representative images of emerging Oct4:GFP+ colonies from blocked cells following treatment with CK-666 or together with ROCKi. Scale bar: 100 μm.

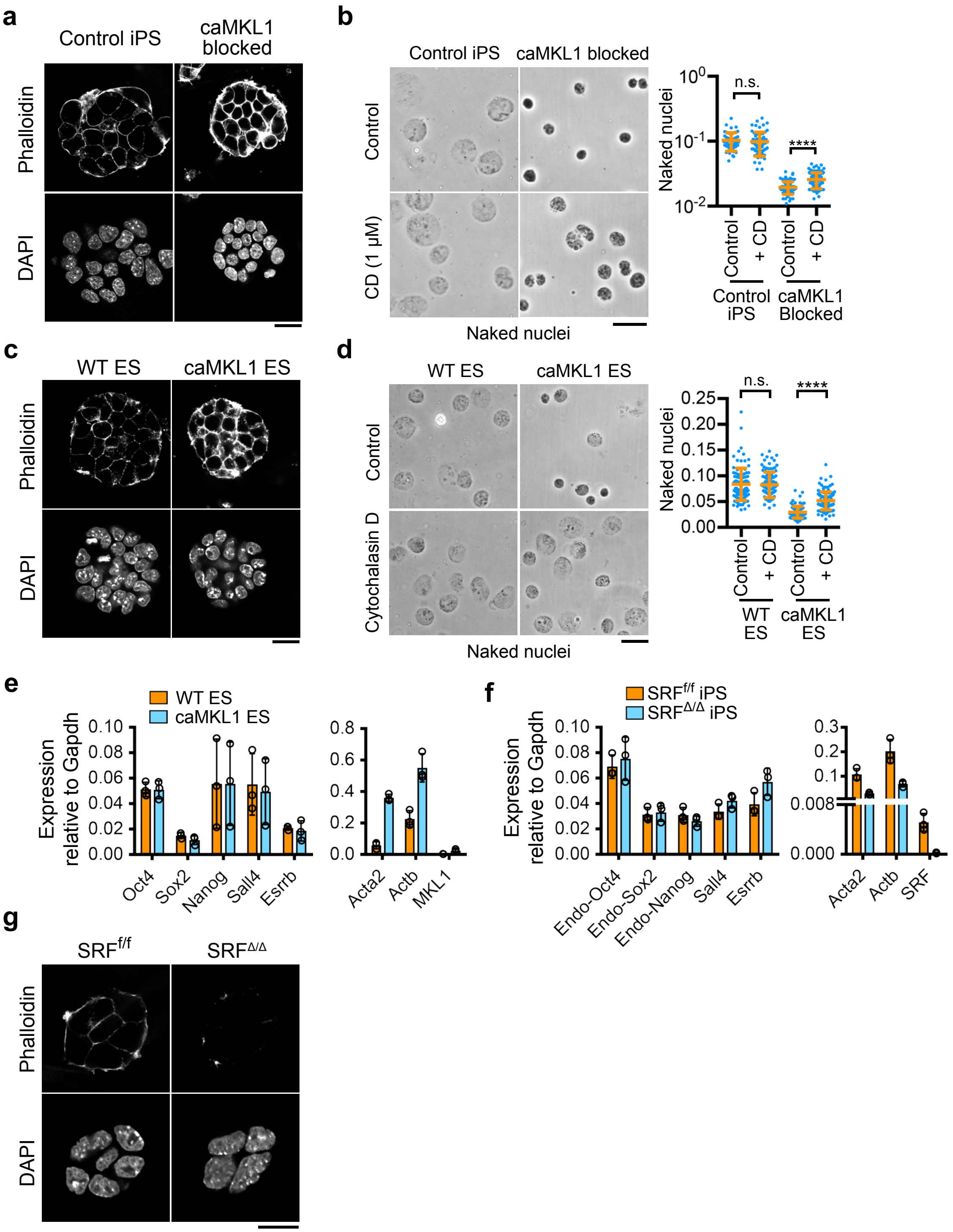

a
Control iPS
caMKL1 blocked
Phalloidin
DAPI
b
Control iPS
caMKL1 blocked
Control
CD (1 µM)
Naked nuclei
n.s.
****
Naked nuclei
Control
+ CD
Control
+ CD
Control iPS
caMKL1 Blocked
c
WT ES
caMKL1 ES
Phalloidin
DAPI
d
WT ES
caMKL1 ES
Control
Cytochalasin D
Naked nuclei
n.s.
****
Naked nuclei
WT ES
caMKL1 ES
e
WT ES
caMKL1 ES
Expression relative to Gapdh
Oct4
Sox2
Nanog
Sall4
Esrrb
Acta2
Actb
MKL1
f
SRF$^{f/f}$ iPS
SRF$^{\Delta/\Delta}$ iPS
Expression relative to Gapdh
Endo-Oct4
Endo-Sox2
Endo-Nanog
Sall4
Esrrb
Acta2
Actb
SRF
g
SRF$^{f/f}$
SRF$^{\Delta/\Delta}$
Phalloidin
DAPI

Supplementary Figure 6. MKL1/SRF activity modulates nuclear state.

a, Representative images of control iPS cells and caMKL1-blocked cells stained with phalloidin and DAPI. Note the elevated phalloidin+ F-actins present in the blocked cells, which display rounder nuclei. Scale bar: 20 μm.

b, Representative images of naked nuclei from control iPS cells and caMKL1-blocked cells. Cells were treated with or without CD for 2.5 hours before removing the cytoplasm. Nuclei were stained with Trypan blue before imaging. The size of the stained nuclei is quantified on the right. Scale bar: 50 μm. n.s.: non-significant; ****: $P < 0.0001$. Statistics are performed using two-tailed unpaired *t*-test.

c, Representative images of wild type (WT) ES cells and caMKL1-overexpressing ES cells stained with phalloidin and DAPI. Scale bar: 20 μm.

d, Representative images of naked nuclei from WT ES cells and caMKL1-expressing ES cells. Cells were treated with or without CD for 2.5 hours before removing the cytoplasm. Nuclei were stained with Trypan blue before imaging. The size of the stained nuclei is quantified on the right. Scale bar: 50 μm. n.s.: non-significant; ****: $P < 0.0001$. Statistics are performed using two-tailed unpaired *t*-test.

e, **f**, Realtime QPCR analyses of pluripotency gene and cytoskeletal gene expression in WT ES cells and caMKL1-overexpressing ES cells (**e**) , $SRF^{f/f}$ and $SRF^{\Delta/\Delta}$ iPS cells (**f**) . n = 3. Data are mean and s.d. Representative data from three independent experiments are shown.

g, Representative images of $SRF^{f/f}$ and $SRF^{\Delta/\Delta}$ iPS cells stained with phalloidin and DAPI. Scale bar: 20 μm.

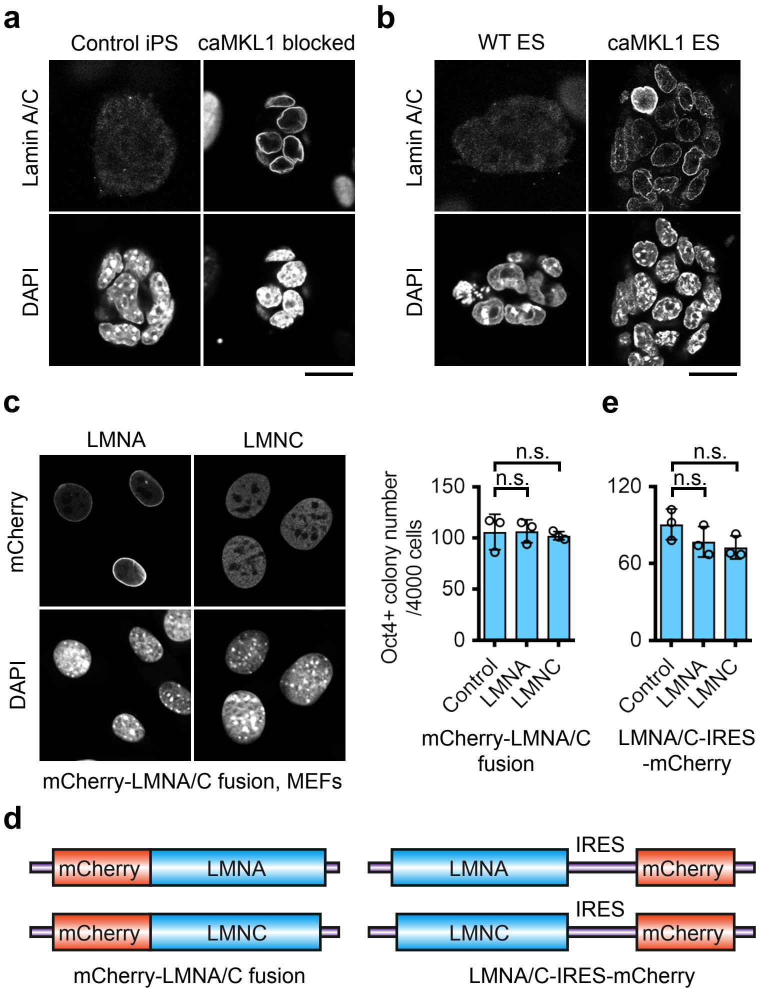

Supplementary Figure 7. Lamin A/C protein is upregulated by MKL1, but overexpression of lamin A/C does not compromise pluripotency activation.

a, **b**, Representative confocal images of indicated cell types following immunofluorescence staining with a lamin A/C-specific antibody. Control iPS cells and caMKL1-blocked cells are shown in **a**; WT and caMKL1-expressing ESCs in **b**. Scale bar: 20 μm.

c, Representative confocal images of reprogrammable MEFs transduced with control vector or retroviral vectors encoding mCherry-LMNA or mCherry-LMNC fusion protein. mCherry signal indicates the proper expression and nuclear localization of the overexpressed proteins (left). Quantification of Oct4:GFP+ colonies are shown on the right. n.s.: non-significant.

d, Schematics of the lamin A/C overexpression constructs.

e, Quantification of Oct4:GFP+ colonies generated from reprogrammable MEFs transduced to express control, LMNA-IRES-mCherry or LMNC-IRES-mCherry. n.s.: non-significant.

n = 3 (**c**, **e**). Data are mean and s.d. Representative data from three independent experiments are shown. Statistics are performed using two-tailed unpaired *t*-test.

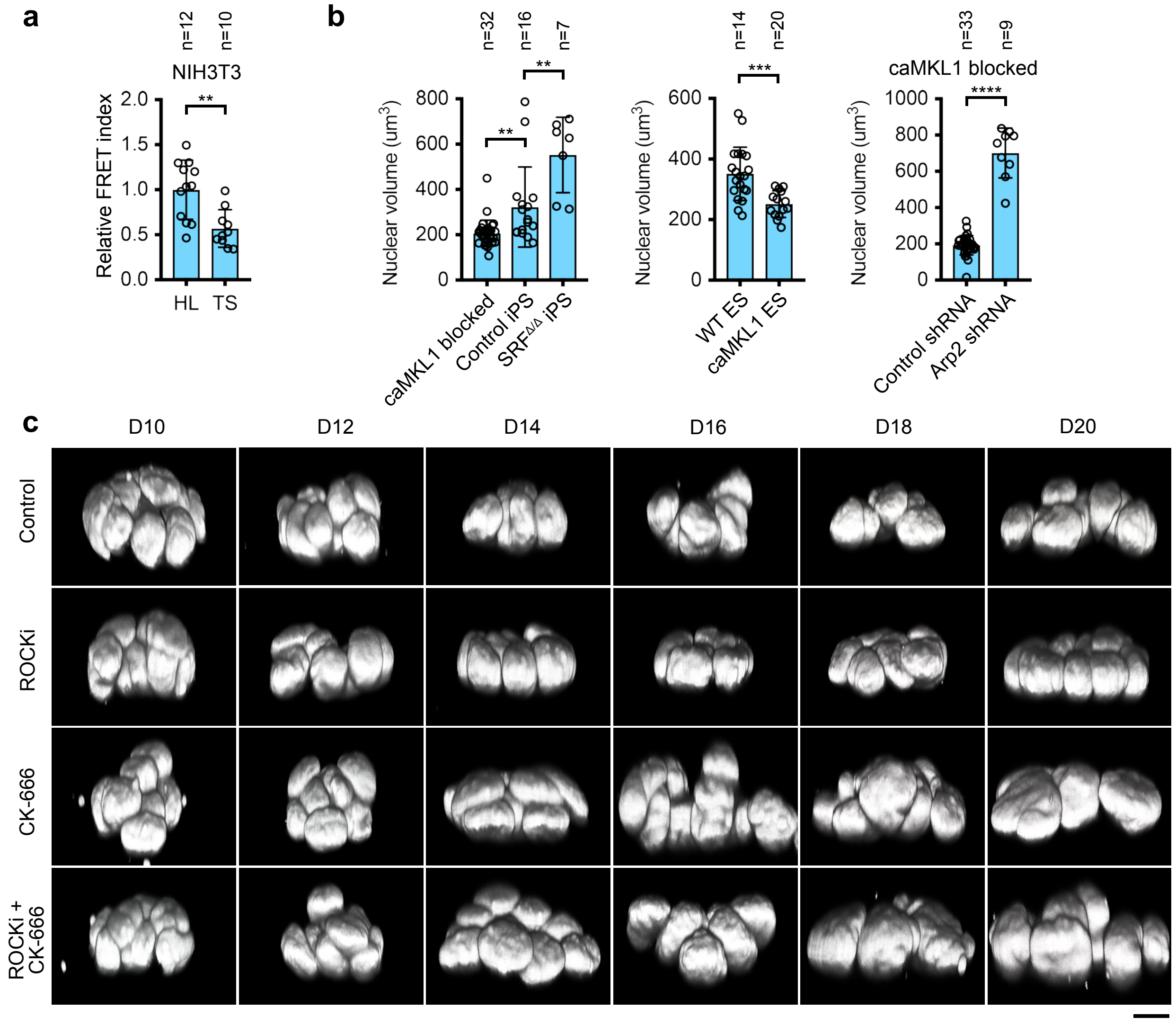

Supplementary Figure 8. Nuclear volume modulation by the actin-MKL1 pathway activity.

a, Validation of the FRET Tension Sensor (TS) and the Headless (HL) control using NIH3T3 cells. In these cells, TS display lower FRET index than HL, confirming the original report by Arsenovic et al., 2016. n denotes the number of nuclei analyzed. **: $P < 0.01$.

b, Quantification of nuclear volumes from 3D reconstructed nuclear images. Data were pooled from multiple independent experiments. n denotes the number of nuclei analyzed. **: $P < 0.01$; ***: $P < 0.001$; ****: $P < 0.0001$. Data are mean and s.d. Statistics are performed using two-tailed unpaired *t*-test (**a**, **b**).

c, Representative 3D nuclear images during the progression toward pluripotency. Reprogrammable MEFs were treated with ROCKi or CK-666, alone or in combination, starting at reprogramming day 10. Images were taken at indicated time points. CK-666 treatment with or without ROCKi showed enlarged nuclei at later time points. Scale bar: 10 μm.

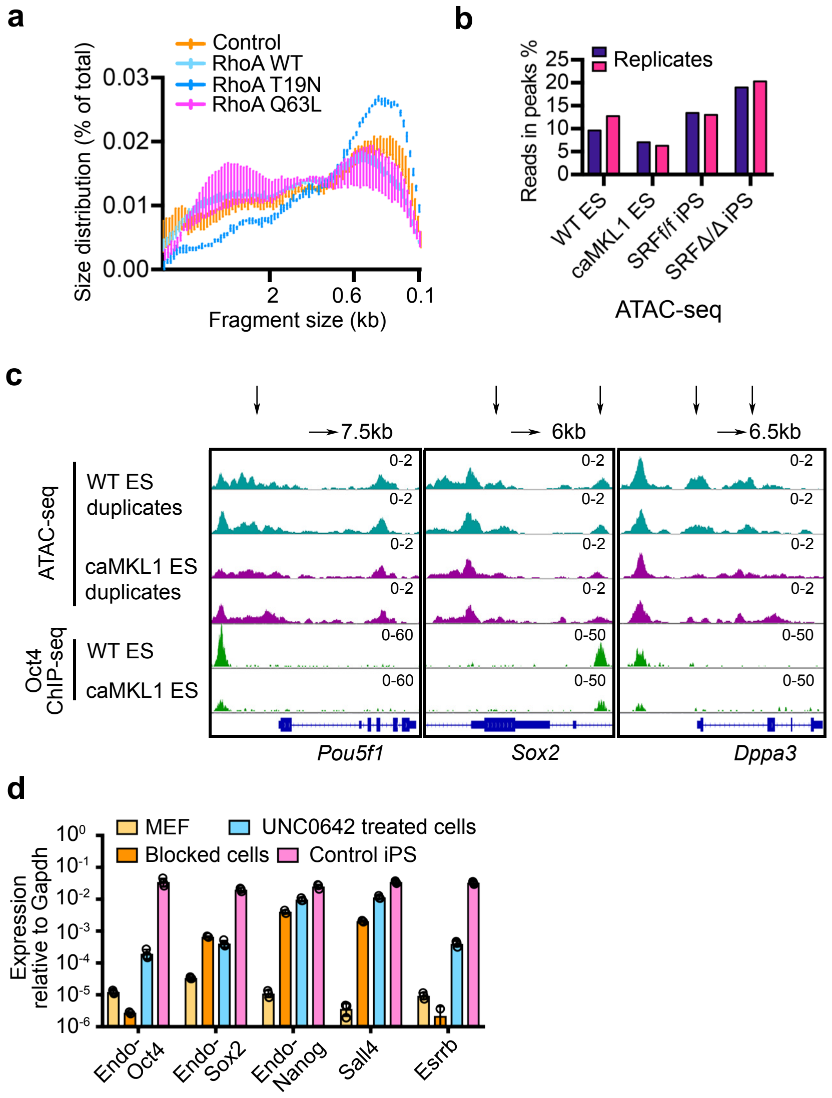

Supplementary Figure 9. MKL1/SRF activity regulates chromatin accessibility.

a, Quantification of the size distribution of DNase I digested DNA fragments from iPSCs ectopically expressing control, RhoA WT, RhoA T19N (dominant negative) or RhoA Q63L (constitutively active). Cells expressing RhoA T19N display more abundant small fragments, while cells expressing Q63L display more large fragments. Mean fragment sizes from triplicate samples are shown.

b, Percentage of reads that are mapped to distinct peaks detected by ATAC-seq. Each sample has biological duplicates.

c, Genome browser screenshots of ATAC-seq data and Oct4 ChIP-seq data from WT ES cells and caMKL1-overexpressing ES cells. Representative pluripotency regions (*Pou5f1*, *Sox2*, *Dppa3*) are shown. Arrows denote genomic regions that display different accessibility in different cell types.

d, Realtime QPCR analyses of pluripotency gene expression in blocked cells following treatment with UNC0642. Their levels in MEFs, untreated caMKL1-blocked cells and control iPS cells are plotted as controls. n = 3. Data are mean and s.d. Representative data from three independent experiments are shown.

Supplementary Table 1

Realtime PCR primers for mRNA		
Primer Name	Forward 5'-3'	Reverse 5'-3'
Acta2	TGAAGCCCAGAGCAAGAGAGG	CGTTATAGAAAGAGTGGTGCCAGA
Actb	TTCTTTGCAGCTCCTTCGTT	ATGGAGGGGAATACAGCCC
Actc1	GAGCTGTCTTCCCGTCCATC	GCTCTGGGCTTCATCACCTAC
Actg2	ACAGGATGCAGAAGGAAATCACA	TGCTGGAAGGTGGAGAGAGAG
Tagln	GCTACTCTCCTTCCAGTCCACA	CCTCCAGCTCCTCGTCATACTT
MKL1	CCTGCTCCCACACTCATCAA	GGTGGTACTTGAGCTTCTTCACC
MKL2	CAGCCTCAGCCAGTCAGAAA	TGGATTGAAGAGCAGGGTTAGAG
SRF	ACCGTGCAGATCCCTGTCTC	CATTCACTCTTGGTGCTGTGG
Endo-Oct4	TCTTTCCACCAGGCCCCCGGCTC	TGCGGGCGGACATGGGGAGATCC
Endo-Sox2	TAGAGCTAGACTCCGGGCGATGA	TTGCCTTAAACAAGACCACGAAA
Nanog	AAATCCCTTCCCTCGCCATC	TTTGGGACTGGTAGAAGAATCAGG
Sall4	GTGTCTCAGCAAGTGTCCGTGT	GCATGAGGTAGCTTGGCTTGTT
Esrrb	GATCGGGAGCTTGTGTTCCTC	AGGCGAGAGTGTTCCTCATCC
Fmn2	AATCAGGACAGAGTGGCTAGGA	AAATAAGCGAAGGGCTGGA
Myh11	CAGAAGGCTCAGACCAAAGAA	CAGAATGCCCAGGAAGGAG
Arp2	TTGGTGTTGCTGAACTGCTTT	TTTAAGCTCTCGTTCCAACCTC
Arp3	ACCTCCACTGAATACTCCAGAAA	GGATGCAGCTAAGGCAAGAA
Sun1	CTACCCAACCACATTCACCA	CCTCTTCTTGATACTCCGTTTCC
Sun2	TTCCTTCTGCTCTTGCTACTCC	ACACCTCTGGCTGCTTCCT
Realtime PCR primers for genomic DNA (ChIP)		
Genomic loci	Forward 5'-3'	Reverse 5'-3'
Oct4 enhancer	CAGGGAGGTTGAGAGTTCTGG	AGATTAAGGAAGGGCTAGGACGA
Gapdh promoter	GAAATGAGAGAGGCCCAGCTAC	TTTATAGGAACCCGGATGGTG
shRNA targeting sequences		
Gene name	Targeting sequence 5'-3'	
MKL1	ATCCTCACTGTGACCAATAAG	
Fmn2	ACGCCAAGTCTCTCGACAAAC	
Myh11	GCCTGCATTCTCATGATCAAA	
Arp2	GCCAGGTTTGAGTCCTTCAAA	
Arp3	GCAGATGTAGAAGAGAGCTAA	
Sun1	AGGCTATTGATTCGCACATTA	
Sun2	TCGGATCTTCCTCAGGCTATT	

Supplementary Methods

Cell culture

MEFs and 293T cells were cultured in DMEM (Gibco, 11995) supplemented with 10% heat-inactivated FBS (Gibco) and 1× Penicillin-Streptomycin-Glutamine (PSG, Gibco). iPS cells and ES cells were cultured in DMEM supplemented with 15% FBS (Hyclone), 0.1 mM non-essential amino acid (NEAA, Gibco), 1× PSG, 0.1 mM β-mercaptoethanol, and 1000 U/mL murine leukemia inhibitory factor (LIF, Millipore). Feeder cells were obtained by irradiating P5-P6 MEFs. Mature iPS cells and ES cells were maintained on feeder layers.

RNA extraction, Reverse Transcription and qPCR

Total RNA was extracted with Trizol® reagent (Invitrogen) and reverse transcribed with the SuperScript® III First-Strand Synthesis System (Invitrogen). Quantitative real-time PCR was performed using the iQ™ SYBR® Green Supermix (Bio-Rad) on a Bio-Rad CFX96.

Chromatin immunoprecipitation (ChIP)

Cells were trypsinized, resuspended, and crosslinked with 1% formaldehyde (Sigma-Aldrich) for 5 min at room temperature (RT). For blocked cells, iPS cells or ES cells, feeder cells were removed before crosslinking. Crosslinking was stopped by adding glycine to a final concentration of 125mM, and cells were incubated at RT for 5 min. Crosslinked cells were lysed in lysis buffer (50 mM Tris-HCl, pH 8.0, 10 mM EDTA, pH 8.0, 1% SDS) on ice for 10 min, and then subject to sonication with a Biorupter (Diagenode) to fragment the chromatin. Sonication product was centrifuged and the supernatant was collected and diluted by dilution

buffer (16.7 mM Tris-HCl, pH 8.0, 1.2 mM EDTA, pH 8.0, 167 mM NaCl, 1.1% Triton X-100, 0.01% SDS), followed by incubation with antibodies at 4 °C overnight. Chromatin-antibody immune-complexes were then incubated with Dynabeads® protein G (ThermoFisher, 10003D) for 2-5 hours, and precipitated by applying the magnetic bar. Chromatin-antibody-protein G Dynabeads® complexes were sequentially washed once, for 5 min each, with low salt buffer (20 mM Tris-HCl, pH 8.0, 150 mM NaCl, 2 mM EDTA, pH 8.0, 1% Triton X-100, 0.1% SDS), high salt buffer (20 mM Tris-HCl, pH 8.0, 500 mM NaCl, 2 mM EDTA, pH 8.0, 1% Triton X-100, 0.1% SDS), LiCl wash buffer (50 mM Tris-HCl, pH 8.0, 250 mM LiCl, 1 mM EDTA, pH 8.0, 1% NaDOC, 1% NP-40), then then washed twice with TE buffer (10 mM Tris-HCl, pH 8.0, 1 mM EDTA, pH 8.0) followed by elution twice with elution buffer (1% SDS, 0.1 M $NaHCO_3$) for 15 min at RT. Precipitated chromatin was added with 5 M NaCl (9 μL for 200 μL eluate), 1 M Tris-HCl (9 μL for 200 μL eluate), 0.5 M EDTA (4.5 μL for 200 μL eluate), and RNase A (20 μg/mL), and incubated at 37 °C for 0.5 hour, followed by de-crosslinking at 65 °C overnight. Protein was removed by adding with Proteinase K (20 μg/mL) and digesting at 55 °C for another 2 hours. Precipitated DNA was then purified with the Gel Extraction Kit (Qiagen).

ATAC-seq

50,000 cells were used per reaction for each cell type. Cells were washed in PBS and lysed using 100 μL cold lysis buffer (10 mM Tris-HCl, pH 7.4, 10 mM NaCl, 3 mM $MgCl_2$, 0.1% IGEPAL CA630). Nuclei were centrifuged at 1500 rpm for 5 min, and resuspended with the transposase reaction mix (25 μL 2× TD buffer, 2.5 μL transposase (illumina FC121-1030) and 22.5 μL nuclease-free water). The transposition reaction was performed at 37 °C for 30 min. After transposition, DNA fragments were purified with Qiagen MinElute Kit. Libraries were pre-

amplified for 5 PCR cycles using KAPA HiFi HotStart ReadyMix PCR kit (KK2601/KK2602) (25 μL KAPA HiFi 2× Mix, 24 μL of Tagmented DNA, 1 μL primer mix), followed by Qiagen MinElute Kit purification. Primers were used as listed in Buenrostro et al 2013. Libraries were tested with QPCR to determine additional application cycles. A final round of amplification was performed, and libraries were purified with Qiagen MinElute Kit before sequencing.

AP staining

AP staining was performed using the AP staining kit from StemGent (00-0055).

Trypan blue staining of naked nuclei

Isolated nuclei were kept in isotonic buffer (RSB buffer) on ice until staining and imaging. Five minutes before imaging, nuclei suspension was mixed 1:1 with trypan blue (ThermoFisher Scientific, 15250-061) and loaded onto a hemacytometer for imaging. Images were captured using an inverted microscope with a 20× objective.

Supplementary References

Guo, S. *et al.* Nonstochastic reprogramming from a privileged somatic cell state. *Cell* **156**, 649-662, doi:10.1016/j.cell.2014.01.020 (2014).

Arsenovic, P. T. *et al.* Nesprin-2G, a Component of the Nuclear LINC Complex, Is Subject to Myosin-Dependent Tension. *Biophys J* **110**, 34-43, doi:10.1016/j.bpj.2015.11.014 (2016).

Buenrostro, J. D., Giresi, P. G., Zaba, L. C., Chang, H. Y. & Greenleaf, W. J. Transposition of native chromatin for fast and sensitive epigenomic profiling of open chromatin, DNA-binding proteins and nucleosome position. *Nat Methods* **10**, 1213-1218, doi:10.1038/nmeth.2688 (2013).
