# Supplementary material for: MKL1-actin pathway restricts chromatin accessibility and prevents mature pluripotency activation
Source: Nat Commun. 2019 Apr 12;10:1695. doi: 10.1038/s41467-019-09636-6 (PMC6461646; doi:10.1038/s41467-019-09636-6)
Supplement: Supplementary file 2 — Reporting Summary [file 41467_2019_9636_MOESM2_ESM.pdf]

nature research

Corresponding author(s): Shangqin Guo

Reporting Summary

Nature Research wishes to improve the reproducibility of the work that we publish. This form provides structure for consistency and transparency in reporting. For further information on Nature Research policies, see Authors & Referees and the Editorial Policy Checklist.

Statistical parameters

When statistical analyses are reported, confirm that the following items are present in the relevant location (e.g. figure legend, table legend, main text, or Methods section).

n/a	Confirmed	
☐	☒	The exact sample size (n) for each experimental group/condition, given as a discrete number and unit of measurement
☐	☒	An indication of whether measurements were taken from distinct samples or whether the same sample was measured repeatedly
☐	☒	The statistical test(s) used AND whether they are one- or two-sided<br>*Only common tests should be described solely by name; describe more complex techniques in the Methods section.*
☒	☐	A description of all covariates tested
☒	☐	A description of any assumptions or corrections, such as tests of normality and adjustment for multiple comparisons
☒	☐	A full description of the statistics including central tendency (e.g. means) or other basic estimates (e.g. regression coefficient) AND variation (e.g. standard deviation) or associated estimates of uncertainty (e.g. confidence intervals)
☒	☐	For null hypothesis testing, the test statistic (e.g. F, t, r) with confidence intervals, effect sizes, degrees of freedom and P value noted<br>*Give P values as exact values whenever suitable.*
☒	☐	For Bayesian analysis, information on the choice of priors and Markov chain Monte Carlo settings
☒	☐	For hierarchical and complex designs, identification of the appropriate level for tests and full reporting of outcomes
☒	☐	Estimates of effect sizes (e.g. Cohen's d, Pearson's r), indicating how they were calculated
☐	☒	Clearly defined error bars<br>*State explicitly what error bars represent (e.g. SD, SE, CI)*

Our web collection on statistics for biologists may be useful.

Software and code

Policy information about availability of computer code

Data collection	N/A
Data analysis	N/A

For manuscripts utilizing custom algorithms or software that are central to the research but not yet described in published literature, software must be made available to editors/reviewers upon request. We strongly encourage code deposition in a community repository (e.g. GitHub). See the Nature Research guidelines for submitting code & software for further information.

Data

Policy information about availability of data

All manuscripts must include a data availability statement. This statement should provide the following information, where applicable:

- Accession codes, unique identifiers, or web links for publicly available datasets
- A list of figures that have associated raw data
- A description of any restrictions on data availability

All genomics data, including RNA-seq, ChIP-sec and ATAC-seq data that support the findings of this study are deposited in public databases with the GEO accession code provided.

Field-specific reporting

Please select the best fit for your research. If you are not sure, read the appropriate sections before making your selection.

☒ Life sciences ☐ Behavioural & social sciences ☐ Ecological, evolutionary & environmental sciences

For a reference copy of the document with all sections, see nature.com/authors/policies/ReportingSummary-flat.pdf

Life sciences study design

All studies must disclose on these points even when the disclosure is negative.

Sample size	Conforming to the standard practice adopted by the field, based on the usual/expected variations and signal strength
Data exclusions	No data were excluded
Replication	All observations reported have been repeated and reproducible
Randomization	N/A
Blinding	The FRET analyses were done blinded between our lab and the collaborating lab. Other experiments were not blinded because most of the work were conducted by the first author, where all samples have to be cleared designated.

Reporting for specific materials, systems and methods

Materials & experimental systems

n/a	Involved in the study
☐	☒ Unique biological materials
☐	☒ Antibodies
☐	☒ Eukaryotic cell lines
☒	☐ Palaeontology
☐	☒ Animals and other organisms
☒	☐ Human research participants

Methods

n/a	Involved in the study
☐	☒ ChIP-seq
☐	☒ Flow cytometry
☒	☐ MRI-based neuroimaging

Unique biological materials

Policy information about availability of materials

Obtaining unique materials	The Nesprin 2G Tension Sensor and Headless control are available from Addgene. All relevant mouse strains, described in detail in accompanying supplemental information, are available from Jackson Lab.

Antibodies

Antibodies used	these information are included in the accompanying supplementary information
Validation	all antibodies have been validated by the vendor. In the case of the custom made MKL1 antibody, the validation information can be found in this publication Willer et al., 2017.

Eukaryotic cell lines

Policy information about cell lines

Cell line source(s)	derived from primary mouse fibroblasts
Authentication	the "caMKL1 blocked cells " are not authenticated. We will establish a profile to allow future authentication.
Mycoplasma contamination	Cell lines were not tested for mycoplasma because they are primary mouse cells.

Commonly misidentified lines (See ICLAC register) | N/A

Animals and other organisms

Policy information about studies involving animals; ARRIVE guidelines recommended for reporting animal research

Laboratory animals	details are provided in the accompanying supplemental information
Wild animals	N/A
Field-collected samples	N/A

ChIP-seq

Data deposition

☒ Confirm that both raw and final processed data have been deposited in a public database such as GEO.

☒ Confirm that you have deposited or provided access to graph files (e.g. BED files) for the called peaks.

Data access links *May remain private before publication.*	Provided by GEO access code.
Files in database submission	Provided by GEO access code.
Genome browser session (e.g. UCSC)	Provided by GEO access code.

Methodology

Replicates	details are provided in the accompanying supplemental information
Sequencing depth	details are provided in the accompanying supplemental information
Antibodies	details are provided in the accompanying supplemental information
Peak calling parameters	details are provided in the accompanying supplemental information
Data quality	details are provided in the accompanying supplemental information
Software	details are provided in the accompanying supplemental information

Flow Cytometry

Plots

Confirm that:

☒ The axis labels state the marker and fluorochrome used (e.g. CD4-FITC).

☒ The axis scales are clearly visible. Include numbers along axes only for bottom left plot of group (a 'group' is an analysis of identical markers).

☒ All plots are contour plots with outliers or pseudocolor plots.

☒ A numerical value for number of cells or percentage (with statistics) is provided.

Methodology

Sample preparation	Cells were made into single cell suspension by trypsinization and filtering
Instrument	BD LSRII, BD Aria
Software	Flowjo and Diva
Cell population abundance	details included with figure
Gating strategy	included with figure

☒ Tick this box to confirm that a figure exemplifying the gating strategy is provided in the Supplementary Information.
