# Supplementary material for: MKL1-actin pathway restricts chromatin accessibility and prevents mature pluripotency activation
Source: Nat Commun. 2019 Apr 12;10:1695. doi: 10.1038/s41467-019-09636-6 (PMC6461646; doi:10.1038/s41467-019-09636-6)
Supplement: Supplementary file 3 — Description of Additional Supplementary Files [file 41467_2019_9636_MOESM3_ESM.pdf]

Description of Additional Supplementary Files

Supplementary Data 1.

Differential gene expression by mRNA-seq between control- and caMKL1 expressing cells at two time points.

Supplementary Data 2.

Differentially bound genomic regions between control iPSC and caMKL1-blocked cells as detected by SRF or Oct4 ChIP-seq.

Supplementary Data 3.

Differential chromatin accessibility as detected by ATAC-seq in pluripotent stem cells of indicated genotypes.
